# How do women experience a false-positive test result from breast screening? A systematic review and thematic synthesis of qualitative studies

**DOI:** 10.1038/s41416-019-0524-4

**Published:** 2019-07-23

**Authors:** Hannah Long, Joanna M. Brooks, Michelle Harvie, Anthony Maxwell, David P. French

**Affiliations:** 1 Manchester Centre for Health Psychology, School of Health Sciences, University of Manchester, Manchester, M13 9PL United Kingdom; 2 Nightingale Centre, Manchester University NHS Foundation Trust, Manchester, M23 9LT United Kingdom; 3 Division of Informatics, Imaging & Data Sciences, School of Health Sciences, University of Manchester, Manchester, M13 9PT United Kingdom

**Keywords:** Cancer screening, Human behaviour, Psychology

## Abstract

**Background:**

This is the first review to identify, appraise and synthesise women’s experiences of having a false-positive breast screening test result.

**Methods:**

We systematically searched eight databases for qualitative research reporting women’s experiences of receiving a false-positive screening test result. Two reviewers independently screened articles. Eight papers reporting seven studies were included. Study quality was appraised. Data were thematically synthesised.

**Results:**

Women passively attended screening in order to prove their perceived good health. Consequently, being recalled was unexpected, shocking and disempowering: women felt without options. They endured great uncertainty and stress and sought clarity about their health (e.g. by scrutinising the wording of recall letters and conversations with healthcare professionals). Their result was accompanied by relief and welcome feelings of certainty about their health, but some received unclear explanations of their result, contributing to lasting breast cancer-related worry and an ongoing need for further reassurance.

**Conclusion:**

The organisation of breast screening programmes may constrain choice for women: they became passive recipients. The way healthcare professionals verbally communicate results to women may contribute to lasting breast cancer-related worry. Women need more reassurance, emotional support and answers to their questions before and during screening assessment, and after receiving their result.

## Background

Breast screening aims to detect and treat early-stage cancer in women, when treatment is less severe and more likely to be successful than for later-stage cancers.^1^ Screening has the potential to reduce breast cancer mortality, but it is also associated with unintended harms, such as overdiagnosis, overtreatment, false-negative and false-positive screening test results.^1,2^ A false-positive screening test result is said to be present when an initial screening mammogram indicates an abnormality, necessitating further diagnostic tests, which indicate cancer is not present.^3^ Under the National Health Service Breast Screening Programme (NHS BSP) in England, 2.2 million women were screened in the 2016–2017 screening period and over 70,000 of them received a false-positive screening test result.^4^

The experience of being recalled and receiving a false-positive screening test result can be distressing for many women.^5–9^ However, the nature of this experience and any psychological impact is not well understood. Systematic reviews have quantified the impact of false-positive screening test results on the women who receive them, but they paint a complex picture with much conflicting evidence.^2,6,9–12^ It is clear that having a false-positive screening test result can lead to short-term breast cancer-related worry, compared to women with normal screening results.^6,9^ One study reported evidence of longer-term breast cancer-related worry at 3 years,^13^ but others have observed that worry abates over time.^14,15^ The evidence suggests that having a false-positive screening test result does not lead to clinical levels of depression or anxiety.^6,9,16^ The impact of receiving a false-positive screening test result on the likelihood of returning for the next routine screening is inconsistent.^6,10,12^ Both the degree of worry and the likelihood of re-attending have been found to be related to the procedures performed when women are recalled for further investigations (a process known as screening assessment). The more invasive procedures, such as needle biopsy, are associated with greater worry and less chance of re-attending the next routine screening appointment.^13,17–19^

There are no peer-reviewed systematic reviews that have included qualitative research of women’s experiences of receiving a false-positive screening test result. One systematic review of qualitative research included both false-positive and false-negative screening test results.^20^ However, this review was not published in a peer-reviewed journal. Further, the review authors did not independently assess the quality of included studies. Cochrane consider quality appraisal to be an essential step in qualitative evidence synthesis, particularly if the findings are to be used to inform subsequent practice and research.^21^ The review found that women who had received false-positive screening test results described feelings of fear and breast cancer-related worry, but were undeterred from future screening. Appropriately, the review authors did not make any practice or research recommendations (e.g. how to reduce the negative impact for women with inaccurate screening results or progress research in this area), but as a result the review’s utility is limited.

Given the inconsistent findings from systematic reviews of quantitative research and the lack of a robust qualitative evidence synthesis, a better understanding of the experience of having a false-positive breast screening test result is needed. Systematic reviews of qualitative studies capture people’s beliefs, perspectives and experiences in both depth (through the qualitative approach) and also breadth (through the integration of studies across different contexts and populations). They offer novel and more comprehensive understanding of phenomena and are increasingly recognised as important in evidence-based decision-making for healthcare and policy.^22–24^

The present review aimed to synthesise women’s experiences of receiving a false-positive breast screening test result. The specific objectives of this review were to (i) systematically identify and appraise existing qualitative research on the experiences, views, and beliefs of women who have received a false-positive screening test result, (ii) use thematic synthesis to synthesise the findings of relevant qualitative research, and (iii) identify directions for future research to improve women’s experiences of having a false-positive screening test result.

## Methods

This systematic review was registered on PROSPERO (CRD42017083404) and is reported according to the PRISMA (Supplementary Materials 1) and ENTREQ statements (Supplementary Materials 2).^23,25^

### Search strategy and eligibility criteria

The following electronic databases were searched: CINAHL, EMBASE, MEDLINE, PsycINFO (each up to 23 January 2018), Web of Science conference proceedings and book citations (up to 5 February 2018), Dissertations Abstracts International, NHS Evidence and Open Grey (each up to 6 February 2018). The ‘Context, How, Issue, Population’ (CHIP) tool was used to formulate the search terms.^26^ The search strategy was designed to be comprehensive and to seek all available studies. The strategy was tailored to the specific indexing language of each database and used medical subject headings (MeSH), other index terms, keywords and appropriate synonyms (Supplementary Materials 3). Search terms were also based on the search strategy of a systematic review of UK-based, quantitative studies on this topic, which had included search terms for qualitative studies.^6^ Databases were searched from 1970 as breast screening for cancer was introduced to Western healthcare systems in the early 1970s. Forward and backward citation searches and hand-searching of the reference lists were conducted for all included papers. EndNote was used to manage retrieved articles.

The first author screened all titles and/or abstracts and a second reviewer screened 30% (k = 1503) of these (99% agreement). The first author read the full texts of all potentially eligible articles and assessed these against the eligibility criteria (Table 1). A second reviewer read the full texts of 50% (k = 11) of these and, following discussion, agreement between the two reviewers was reached.

**Table 1:** Eligibility criteria

Inclusion criteria
(a) Qualitative methodology (i.e. data collection and analysis).
(b) Adult women (aged 18+ years) who have received a false-positive breast screening test result or an abnormal breast screening test result (and are awaiting screening assessment or the associated results).
(c) Mixed samples of adults screened for, or diagnosed with, other cancer types only if it is possible to separately identify those findings related to having a false-positive breast screening test result.
(d) Any country.
(e) Published in English.
Exclusion criteria
(f) Study findings could not be separated in criterion (c).
(g) The sample was all diagnosed with breast cancer (invasive and ductal carcinoma in situ).
(h) Individual case studies.

### Quality appraisal

Study quality was assessed using a modified Critical Appraisal Skills Programme (CASP) tool for qualitative research.^27^ Two papers reported one study, and these were appraised separately as they answered different research questions. Adequately reporting one’s approach to inquiry has been recognised as an indicator of methodological quality in qualitative primary studies,^28^ but the original CASP tool does not have an item related to this. Therefore, the research team included an additional question: ‘are the study’s theoretical underpinnings (e.g. ontological and epistemological assumptions; guiding theoretical framework(s)) clear, consistent and conceptually coherent?’. A fourth response—‘somewhat’—was added for questions answered ‘yes’, ‘no’, or ‘can’t tell’, to indicate when a criterion had been partially addressed but lacked some key elements.

The first author independently appraised the quality of all included studies. Following this, a second and third reviewer independently appraised the quality of one study each (k = 2; 25%) and these decisions were discussed in-team. This process facilitated piloting of the additional question and checked the reliability of quality appraisal decisions. In the synthesis, greater weight was given to findings of studies determined to be of higher quality. The quality appraisal results did not determine study inclusion.

### Data extraction

A standardised data extraction sheet was used to extract research aims, country of origin, sample size, data collection method and data analysis method. The data to be synthesised were the primary study authors’ interpretations of their study findings and all direct quotations from participants. As per Thomas and Harden’s guidelines for thematic synthesis,^29^ all text contained beneath the article heading ‘results’ or ‘findings’ was treated as data, and any findings reported in the abstract.

### Data synthesis

This review followed Thomas and Harden’s method of thematic synthesis.^29^ The first author (a PhD student researcher with a health psychology background) read the full set of papers several times. Two other authors (experienced researchers with expertise in health psychology, cancer screening and qualitative methods) each read a different half of the set of papers. The present approach to inquiry was informed by dialectical pluralism. This approach allows for work undertaken from multiple and often competing paradigms (and underpinned by differing epistemological and ontological assumptions) to be combined into a new, agreed upon whole.^30^

Data analysis involved three main stages. First, the first author undertook inductive *line-by-line coding* of the primary study findings, with regular discussion with other authors. Participant quotations were coded initially (i.e. first-order codes), followed by the primary authors’ narrative and interpretations (i.e. second-order codes). During coding, each line of text is assigned one or more codes that summarises its context and meaning. A preliminary list of codes was developed based on the findings of the highest quality studies. These codes were applied to the findings of studies of medium quality, and new codes added where appropriate. The findings of the lowest quality studies were coded using this list, but no new codes based on these findings were created.

Second, *d* *e* *s* *c* *r* *i* *p* *t* *i* *v* *e* *t* *h* *e* *m* *e* *s* were produced by grouping codes according to their conceptual and descriptive similarities and given overarching labels. Third, *a* *n* *a* *l* *y* *t* *i* *c* *a* *l* *t* *h* *e* *m* *e* *s* were generated by considering the descriptive themes in relation to the review objectives and identifying conceptual links across the descriptive themes, to infer novel interpretations of the primary study findings. Preliminary analytical themes were discussed as a research team on multiple occasions, to reach agreement on the most appropriate thematic structure.

## Results

### Search results

**Fig. 1:**
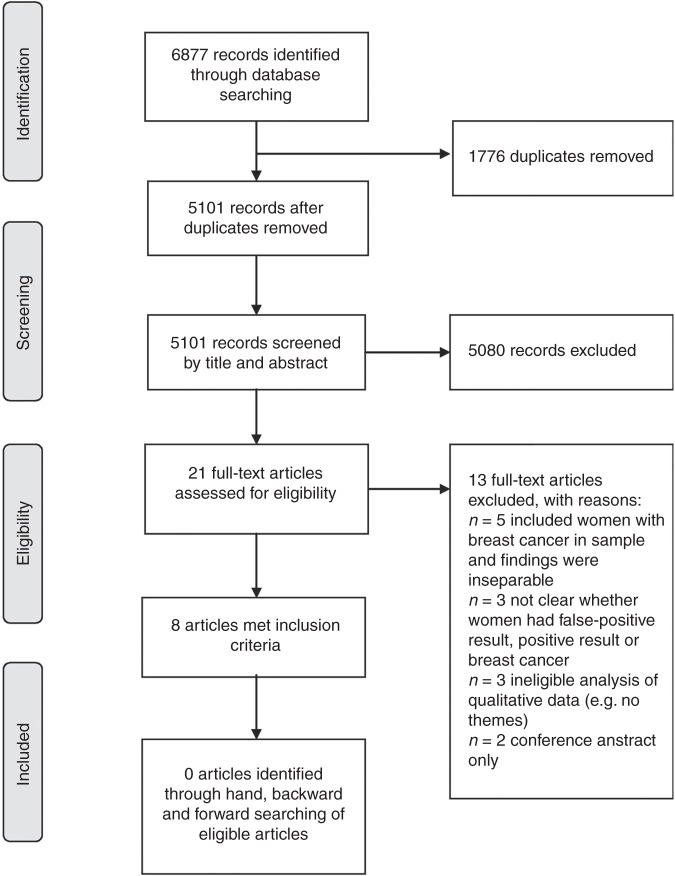
PRISMA flow diagram of study inclusion process

Electronic searches identified 6877 articles (Fig. 1). In total, eight papers, describing seven studies, were eligible for inclusion (Table 1).

### Study characteristics

All papers were published between 2001 and 2015 (Table 2). The eight papers were from six countries: the UK (k = 2), the USA (k = 2), Hong Kong (k = 1), Denmark (k = 1), Norway (k = 1) and Sweden (k = 1). The two papers from the UK were based on the data collected by one study and used different analysis methods to answer different research questions.^7,31^ The primary focus of all studies was to explore women’s perspectives on and experiences of having a false-positive breast screening test result. One study also sought to understand the views of women with false-positive screening test results with the specific purpose of developing improved breast screening services.^31^

**Table 2:** Main characteristics of included papers

Author (year)	Country	Participants	Time since false-positive test result	Data collection	Data analysis
Bolejko et al.^5^	Sweden	*n* = 13 Age range 40–68 year Mean age 51 year	Range: 3–11 month	Semi-structured interviews	Inductive content analysis
Bond et al. (2015a)	UK	*n* = 21 Age range 42–69 year Mean age 59 year	≤1 y *n* = 4 2–4 year *n* = 7 5–7 year *n* = 8 8–10 year *n* = 1 11–13 year *n* = 1 Mean: 4.4 year	Semi-structured interviews	Interpretative phenomenological analysis
Bond et al. (2015b)	UK	*n* = 21 Age range 42–69 year Mean age 59 year	≤1 year *n* = 4 2–4 year *n* = 7 5–7 year *n* = 8 8–10 year *n* = 1 11–13 year *n* = 1 Mean: 4.4 year	Semi-structured interviews	Inductive content analysis
Fielding & Lam^33^	Hong Kong	*n* = 9 Age range 40–62 year	Not reported	Semi-structured interviews	Not explicitly aligned with one method; appears to be thematic
Lindberg et al.^8^	Denmark	*n* = 8 Age range 57–72 year Mean age 65 year	Range: 4–5 year	Semi-structured interviews	Grounded theory
Padgett et al.^34^	USA	*n* = 45 Mean age 52 year	Not explicitly reported; at least 6–8 month	Interviews with open ended questions	Not explicitly aligned with one method; describes grounded theory techniques
Solbjor et al.^32^	Norway	*n* = 8 Age range 50–59 year	Not yet received false positive test result; interviewed the day before screening assessment	Semi-structured interviews at two time points	Not explicitly aligned with one method; appears to be thematic
Thomson & Siminoff^35^	USA	*n* = 40 Age range 40–68 year	<3 month	Interviews with open ended questions and questions about reactions to health guidelines	Directed content analysis

The women included in the primary studies were interviewed at varying time points following screening, ranging from a few days to over 12 years. One study interviewed eight women at two time points: in the interim between receiving a recall letter and attending screening assessment and again after the interviewees received their false-positive breast screening test result.^32^ Two participants were diagnosed with cancer. The findings from the first and second interview data were separately reported. Only findings from the first interview were synthesised in the present review.

### Quality appraisal results

The included studies were of mixed quality, ranging from moderately good^7^ to poor^33,34^ (Supplementary Materials 4). There was often little to no evidence that the authors considered their own role, potential bias or influence in the research design and with participants. Most studies had data analysis issues (e.g. insufficient methodological detail and issues of rigour and trustworthiness). None of the studies adequately reported the study’s ontological and epistemological underpinnings. Two studies were rated as particularly low quality,^33,34^ bringing the trustworthiness of their findings into question. As a result, no new codes were developed based on the data extracted from these studies. The data were used to support the codes generated by analysing the findings of higher quality studies.

### Results of the synthesis

Eight descriptive themes were generated: *p* *e* *r* *c* *e* *p* *t* *i* *o* *n* *s* *o* *f* *h* *e* *a* *l* *t* *h*, *s* *t* *a* *t* *e* *o* *f* *u* *n* *c* *e* *r* *t* *a* *i* *n* *t* *y*, *m* *a* *k* *i* *n* *g* *s* *e* *n* *s* *e* *o* *f* *t* *h* *e* *s* *i* *t* *u* *a* *t* *i* *o* *n*, *b* *e* *i* *n* *g* *‘* *l* *o* *o* *k* *e* *d* *a* *f* *t* *e* *r* *’* *b* *y* *t* *h* *e* *s* *y* *s* *t* *e* *m*, *c* *o* *m* *p* *l* *y* *i* *n* *g* *w* *i* *t* *h* *t* *h* *e* *s* *c* *r* *e* *e* *n* *i* *n* *g* *p* *r* *o* *g* *r* *a* *m* *m* *e*, *b* *r* *e* *a* *s* *t* *c* *a* *n* *c* *e* *r* *s* *p* *e* *c* *i* *f* *i* *c* *w* *o* *r* *r* *i* *e* *s* *a* *n* *d* *a* *n* *x* *i* *e* *t* *i* *e* *s*, *a* *t* *t* *i* *t* *u* *d* *e* *s* *t* *o* *w* *a* *r* *d* *s* *s* *c* *r* *e* *e* *n* *i* *n* *g* *a* *n* *d* *r* *e*-*a* *t* *t* *e* *n* *d* *a* *n* *c* *e*, and *r* *e* *a* *p* *p* *r* *a* *i* *s* *a* *l* *o* *f* *l* *i* *f* *e*. From these descriptive themes, three analytical themes were produced: *e* *x* *p* *e* *c* *t* *a* *t* *i* *o* *n* *s* *f* *o* *r* *t* *h* *e* *i* *r* *h* *e* *a* *l* *t* *h* *a* *n* *d* *s* *c* *r* *e* *e* *n* *i* *n* *g* *e* *x* *p* *e* *r* *i* *e* *n* *c* *e*, *l* *i* *v* *i* *n* *g* *w* *i* *t* *h* *u* *n* *c* *e* *r* *t* *a* *i* *n* *t* *y*, and *r* *e* *s* *t* *o* *r* *a* *t* *i* *o* *n* *o* *f* *t* *h* *e* *h* *e* *a* *l* *t* *h* *y* *s* *e* *l* *f*. Supplementary Materials 5 shows a thematic map of the findings. Author quotes from the primary studies are indicated by use of italics and participant quotes by quotation marks.

### Expectations for their health and screening experience

At the point of invitation, women seemed to perceive the screening programme as a service for healthy women; as a means to prove and evidence their good health, rather than to diagnose breast cancer. Women felt at low, or no, risk of cancer, and willingly accepted the invitation to be screened: “When I got the first invitation to a mammography screening I was looking forward to it very much because I don’t have any illnesses. I’m fit and healthy, nothing is wrong with me, and I haven’t felt any changes or strange sensations at all.”^8^

Women appeared to unquestioningly comply with the screening invitation; attending screening was seen as a conscientious and responsible action. They perceived screening as *part of their health maintenance routine* ^7^ and as an opportunity to be a *health responsible citizen*.^8^ Lindberg et al.^8^ noted: *they* [women] *did not recall having read the enclosed information leaflet, indicating that participation was not a result of an informed, rational choice based on the information provided on the benefits and harms of screening*.

They described welcoming the invitation to be screened and, in some cases, were relieved to be invited: “There was a relief that I was now in the system in a way that meant that I would have regular mammograms.”^7^ Being ‘in the system’ offered women a sense of security and comfort, and they perceived that the role of the service was to look after women: “Someone is keeping an eye on you. Then you can feel safe knowing that nothing is wrong with you.”^8^ Bond et al.^7^ interpreted this as a handing of responsibility for their health, in some measure, over to the NHS, wherein women became passive partakers of assessment procedures.^31^

Having understood screening as an opportunity to receive a clean bill of health, women did not expect to be recalled and described feeling surprised and shocked as a result. They felt they had no option but to undergo the additional tests and to live with the accompanying uncertainty in the interim. Having opted into the process of breast cancer diagnostics, they experienced that there was no way out other than awaiting the follow-up examination.^32^ However, while some described a need for more consultation and shared decision-making, they also reported feeling disempowered by the assessment process: “I think when you’re in the middle of it, you just go along with whatever’s being told… there could have been, uh, more consultation maybe at the beginning of things… so… and I’d have probably still have gone along with it, [biopsy] ‘cause I don’t think I felt empowered not to.”^31^ Women expressed a desire for greater involvement in decisions about how to proceed in assessment and requested choice in the following decisions: (i) a repeat mammogram or ‘watchful waiting’ in place of a biopsy for lesions thought to be benign, (ii) whether to receive biopsy results over the telephone or by post and (iii) a follow-up mammogram for reassurance.^31^

At screening assessment, some women felt well cared-for and supported by the service’s healthcare professionals (HCPs). Other women, in contrast to their expectations, perceived the service and HCPs to be task-focused rather than person-focused: “Sometimes I feel like you been herded around like old cattle, you know, like from one room, then another. And at that particular time when you’re put into a room nothing is really explained to you.”^34^

It may be the case that women have implicit expectations, which are set in part by the screening service. As Bond et al.^7^ explained: *this passivity may be a consequence of the UK programme where the initiative is taken by the NHSBSP who send eligible women an appointment*. The inference is that decisions regarding when and where a screening appointment takes place is decided for eligible women by the programme and thus requires no ‘active part’ from the women, to organise or maintain participation in the service. Thus, the programme structure may set a tone for how women subsequently engage with the service.

### Living with uncertainty

Within and between studies, there was variation in women’s responses to receiving a recall letter, from nonchalance to extreme fear.^7^ Many women reported feeling thrown into a period of uncertainty that was stressful and difficult to live with: “There was a lot of stress. I was stressed out not knowing what it was, and then being too scared to find out what it was and thinking the worse. That it would be cancer.”^34^ The strain of the uncertainty came from: “Just the not knowing, I think. It’s probably just the space in between getting the letter and having the appointment, there’s a space where you… your mind, obviously, is going to play tricks on you and, you know, what if this and that and anxiety. That would have been the worst time.”^7^ As Bolejko et al.^5^ described: *Being in a state of uncertainty, such as not knowing the diagnosis when waiting for examination results, was seemingly unbearable*.

Women felt that they were ‘in limbo’: *they* [women] *remembered that they experienced time as passing slowly, as they waited for the diagnostic procedures and final result*.^8^ For many, this waiting period was characterised by intrusive thoughts and worries about their screening assessment, receiving their results and the possibility of having breast cancer. In all studies, there were women who assumed ‘the worst’ on receipt of a recall letter: “I was just pretty terrified. I mean, my initial thought was ‘Oh, I have breast cancer!’”^35^ Bond et al.^7^ reported: *her self-belief had changed from being a healthy woman to a breast cancer patient in the time it took to read the letter*. Many women reported a fear of breast cancer; they worried about the unknown and potentially catastrophic consequences of having cancer and associated the disease with death: “I was afraid it was cancer. That’s all I could think about. He saw some abnormal findings and made me come in for the second mammogram… I think it [cancer] is a death sentence.”^34^

In the absence of concrete answers, women described anxieties interlaced with coping and attempts to think positively, including looking for reassurance and signs of hope. Some reported an initial fear of having cancer, followed by attempts to be rational and realistic about their risk: “I think about it of course… but I’m not really worried yet. I know things can happen, but at the same time I feel that I need facts to relate to before I really start to worry.”^32^ Some attributed the reason for their recall to potential external factors, such as *a fault with the mammography equipment*.^7^ Table 3 presents additional examples of cues in women’s experiences that were interpreted as reassuring or worrying, before their false-positive screening test result (see Supplementary Materials 6 for contextual detail and supporting evidence from the included studies).

**Table 3:** Signs that women interpreted and analysed to gauge their breast cancer risk

Sign
Their previous screening experiences.
The screening experiences of friends and family.
The presence or absence of a family history of breast cancer.
The wording of the recall letter.
The risk estimates provided in the recall letter.
The location of their screening assessment.
The turnaround time between their recall letter and screening assessment.
The communication with and between HCPs.
The image of their lesion.
Breast self-examination.

### Restoration of the healthy self

Women described feeling great relief when they received their false-positive screening test result, resolving their uncertainty and marking an end to their distress: “I came out feeling great… it’s quite intense, um, so great feeling of relief, yes, and slightly drained as well feeling [laughs], because it is very, yeah, very tense… I was too dazed anyway, to be honest, to take it all in”.^7^ Some women described the experience as “a turning point”^8^ and a “wake up call”^35^ and expressed gratitude and appreciation of life, and of being in good health. For some women, the experience appeared to be life-affirming and prompted self-reflection: “Wow, this is simply amazing! Now I really have to start leading a healthy lifestyle, and I also have to be a better and nicer person.”^8^

Generally, their false-positive screening test result reassured women and restored their perception of being in good health. However, their result was understood in terms of a narrow escape from cancer, rather than an inherent limitation of screening. On the whole, women believed their results were accurate. Some reported feeling particularly reassured when HCPs explained their result to them clearly: “He was ever so… really thorough, I’ve got to say, really put my mind at rest… explained everything to me from start to finish, … and did it in a way… not condescending way, he explained it in a real clear and concise manner, yeah, absolutely brilliant.”^31^

However, others received inconsistent, unclear or inadequate explanations from HCPs of the reason for their recall and result: “I feel confused about what he said to me and he didn’t really answer my questions.”^33^ Unclear explanations left women with residual worries, unanswered questions and feelings of insecurity about their health, contributing to lasting uncertainty about being free of cancer, e.g. ambiguous explanations: “She [consultant] said there was a shadow there, but it wasn’t anything to worry about and [I’m] actually thinking, ‘what is that shadow?’ I don’t think that was ever really quite resolved… at the back of my mind I always thought, ‘I wonder what that shadow was’.”^7^ Consequently, Bond et al.^31^ noted: *there was evidence of an unmet need for information and reassurance that could have been given by a CNS* [Clinical Nurse Specialist] *being available, and known to be available, after as well as before assessment*.

Despite the stress of their experience, women maintained that screening is important and of value and were willing to attend future screening appointments. However, fear of developing breast cancer was often underlying comments regarding future screening. Some women expressed a desire for shorter intervals between screenings: “I thought ‘can you just see me in a year’s time, just tell me that in years’ time, it’s all OK in a year’s time’?”^31^ and “I’m not quite satisfied because he asked me to have mammogram every 2 years but I think it’s not enough. What if there’s something wrong again 1 year later?”^33^ This was particularly the case for those women who experienced lasting uncertainty about being free of cancer, representing a wish for more (and more frequent) reassurance: “I think the fact that you’ve been faced with the possibility that something didn’t look quite right, you’re not quite sure what it was that didn’t look quite right, um, and maybe a screening a year down the line would have been, um, something to… to, you know, relieve any nerves.”^31^ Subsequent screenings presented an opportunity to resolve any lingering doubts about their false-positive screening test result. Some wanted the programme to be extended indefinitely: “I’d want to continue, if I was allowed. Until the day I die. Yes, that’s what I’d prefer.”^8^

Some women expressed more complex attitudes towards re-attendance. For example, some alluded to the decision as being a ‘head versus heart’ dilemma, due to an increased awareness of their susceptibility to breast cancer: “Actually, I don’t want to go for any more mammography, but common sense tells me to; emotionally, I’d much prefer not having to go. I’ve always thought that I couldn’t get BC [breast cancer], but now, common sense tells me otherwise of course.”^5^ Some women described feeling motivated to re-attend because they understood that catching early-stage breast cancer improves their chances of treatment success. Some authors reported on the ‘catch-22’ of screening: *women indicated that screening gave them reassurance and evidence that they were healthy while at the same time raising their anxiety about breast cancer*.^8^ Few women explicitly voiced this issue; one woman expressed hesitations about whether she would actively re-attend when she was no longer in the eligible age-range for screening. She was unsure whether the potential distress of another false-positive screening test result would be worth it, to know she was free of cancer.^7^

## Discussion

### Overview of findings

Three analytic themes were developed to capture how women experience a false-positive screening test result. Women compliantly accepted their screening invitation, keen to have their good health confirmed to them. They were therefore surprised to be recalled and felt compelled to undergo the additional tests. Some women expressed a desire for greater consultation with and support from HCPs regarding their diagnostic options at screening assessment. Overall, women experienced significant stress and a time of high uncertainty. During this time, women described strenuous efforts to understand the reason for their recall and the likelihood of having breast cancer. Such acts (e.g. internalising the experiences of others and scrutinising the wording of recall letters and conversations with HCPs) were reassuring for some but produced further worry for others. Seeking more information was often an active process of interpreting and overanalysing every related message. Receiving the false-positive screening test result generally brought women great relief. However, inadequate explanations from HCPs left some women in doubt about the circumstances of their false-positive screening test result and their health. Some women felt positive about re-attending screening, whereas others saw it as a necessary responsibility, but one they did not relish, and others wanted more frequent reassurance.

### Relevance to existing literature

Previous research indicates that having a false-positive screening test result can lead to short- and longer-term breast cancer-related worry.^6,9^ In support, this review found that women expressed a number of worries related to breast cancer, which were felt before their screening assessment and beyond their false-positive screening test result. The present review also offers novel insight: women’s intentions to re-attend appeared to be linked to seemingly new concerns about their breast cancer risk and lingering doubts about their false-positive screening test result. Moreover, the findings suggest that the structure of breast screening programmes engenders passive participation from women who are seeking a certificate of good health. Of note, this notion was particularly prominent in the findings from the European studies, where women are routinely invited to screening, but not in the findings from the North American studies, where participation in screening is ‘opt-in’. Being recalled and receiving a false-positive screening test result challenges their beliefs about their health and explains why worry is so present for these women. It is possible that the degree of breast cancer-related worry may be poorly captured by quantitative measures, given its prevalence in these qualitative studies. It is also possible that women who have experienced significant worry due to a false-positive screening test result are more likely to participate in related research.

### Strength and limitations

Explicit, systematic and reproducible methods were used to identify eligible studies, including relevant grey or unpublished literature, and a thorough and comprehensive quality appraisal was conducted. Thematic synthesis was conducted according to published methodological guidelines. The review protocol was registered on PROSPERO and adheres to PRISMA and ENTREQ reporting standards. This review is the first systematic review and thematic synthesis of women’s experiences of having a false-positive breast screening result and has identified a number of directions for future research.

The review has some limitations. Only English language studies were included. The synthesis relied on the primary study authors’ interpretations and published quotes and it not possible to know how representative these are of the data as a whole, which may be problematic as the primary studies were of mixed quality. It should be noted that the data synthesised from Solbjor et al.^32^ were of women interviewed between receiving their recall letter and attending screening assessment. Therefore, this data pertain to the experience of being recalled for an inconclusive screening, not specifically to the receipt of a false-positive screening test result. However, there did not appear to be any substantial differences between women’s experiences reported by this study compared with others. Two of the included studies^5,8^ purposively sampled women who showed evidence of breast cancer-related worry indicated by scores on the Consequences of Screening in Breast Cancer questionnaire.^36^ It is likely that the views of women who were less negatively affected by their experience are underrepresented in these studies, and possibly in the overall review findings.

### Implications for practice

The findings have implications for the sensitive care of women who have been recalled. It would be helpful for screening units to be better prepared to deal with the significant distress and uncertainty felt by women. Current practice guidelines in the UK advise that the availability of a clinical nurse specialist (CNS) at screening assessment would provide a vital source of support for women, helping them to cope with the uncertainty of the situation and to answer any questions they may have about the assessment process.^37^ All leaflets and letters sent to women recalled for further investigations should detail how they can contact a CNS for information and support. Furthermore, women wanted the recall letter to explain that they could bring a friend or relative to screening assessment for support (Supplementary Materials 6).

It is also worth considering women’s access to and engagement with screening materials. It remains important that the provision of screening materials by screening centres is routine and consistent. For example, in the UK, it is current practice for screening centres to provide women with a standardised screening information leaflet with every incident and prevalent screening invitation. However, it is not known how well women engage with the screening materials upon each screening invitation. It may also be important to consider whether alternative means to access the necessary information, including in different language translations, is possible and, if so, made clear.

### Implications for research

The present findings suggest that women have preferences for how their false-positive screening test results are communicated to them. Women responded well to clear, thorough and unambiguous explanations of their results, as well as the opportunity to ask questions. In the UK, the 2016 NHS BSP guidelines for communicating results advise that false-positive screening test results should be given to women in person (as opposed to by telephone or post),^38^ but the present findings indicate that it is more complex than this. It is not currently known how to best communicate false-positive screening test results, both at screening assessment and, for those who undergo biopsy, at the results appointment. This warrants further research, particularly if there is the potential to reduce lasting breast cancer-related worry by improving the communication of results.

Some women experienced lasting breast cancer-related worry, but for how long or to what degree is unclear. There is need for longitudinal quantitative and qualitative studies to examine the impact of false-positive screening test results on lasting worry. However, current quantitative measures of worry may not adequately capture the persistent and intrusive nature of this experience. It will be useful to collect information on, for example, the reason for the recall, the procedures performed at screening assessment and the nature of the false-positive screening test result, to see whether these may be contributing factors to lasting worry and areas for possible research intervention. Further, worry about breast cancer may be a key motivation for screening re-attendance. The extent to which this is the case should be explored, as well as the reverse scenario: whether such worry could be a deterrent to re-attendance. This may be particularly relevant for countries observing a decline in, or wanting to increase, screening uptake. In the UK, uptake currently stands at 71.1% of the total number of women invited but is falling year on year.^39^

Given that some women engaged in self-reflection about life and their health following their false-positive screening test result, it may be worth exploring the extent to which women deem such a time to be an appropriate and acceptable ‘teachable moment’, to encourage women to make lifestyle changes to reduce their risk of breast cancer. The results of the Promoting Early Presentation trial provide support for the potential of teachable moments in breast screening.^40^ In this study, older women attending their final routine screening appointment received a brief intervention that increased breast cancer awareness at three years. Further research with the subgroup of women who have received a false-positive breast screening test result may usefully explore whether they would be receptive to health messages in this time. It is plausible that attitudes could differ depending on whether their perceptions of being in good health have been re-confirmed or challenged without resolution.

Women’s initial decisions to attend screening did not appear to be based on prospective ‘weighing up’ of the harms and benefits of screening to make an informed choice, but instead on a desire to substantiate their good health and to act conscientiously. Moreover, women did not appear to retrospectively perceive the reasons for having a false-positive screening test result in terms of the inevitable limitations of screening, but as a close call with breast cancer and, for some, a cause for lasting health concern. When considered together, these findings have implications for the concept of informed choice, which is explicitly encouraged by Western healthcare systems.^41^ In recent years, screening programmes in Europe have made significant efforts to improve the information that is provided to women. For example, in the UK, the NHS BSP has revised the information leaflet sent to women invited for screening and the letters of correspondence throughout the process.^42^ However, there is no guarantee that improved information is sufficient to impact on women’s decision-making processes, particularly when systems are set up to reduce choice.^43^ The present findings suggest that current practices may not be resulting in decisions based on appraisal of the harms and benefits of screening.

## Conclusions

Receiving a false-positive screening test result is a stressful experience for women, characterised by uncertainty and worry about having breast cancer. There is the potential for worry to last, and this may influence women’s attitudes towards future screening and be a factor in their decisions to re-attend. The way that breast screening programmes are organised and delivered may hinder informed decision-making. Future research could examine how to best communicate false-positive screening test results and the potential for this event to motivate women to adopt behaviours that reduce their risk of breast cancer.

## Supplementary information

Supplementary materials, tables, figures, and legends

## Data Availability

The data extracted and synthesised in the present review are available from the corresponding author upon request.

## References

[1] Independent UK (2012). Panel on Breast Cancer Screening. The benefits and harms of breast cancer screening: an independent review. The Lancet.

[2] Gøtzsche PC, Jørgensen KJ (2013). Screening for breast cancer with mammography. Cochrane Database Syst. Rev..

[3] World Health Organisation (WHO) International Agency for Research on Cancer. *Breast cancer screening*. *International Agency for Research on Cancer*. (WHO, Lyon, France, 2002).

[4] NHS Digital. Breast Screening Programme. https://files.digital.nhs.uk/pdf/m/f/breast_screening_programme__england__2016-17_-_report__v2.pdf, cited 2019 Dec 16, [2017]

[5] Bolejko A, Zackrisson S, Hagell P, Wann‐Hansson C (2014). A roller coaster of emotions and sense–coping with the perceived psychosocial consequences of a false‐positive screening mammography. J. Clin. Nursing.

[6] Bond M, Pavey T, Welch K, Cooper C, Garside R, Dean S (2013). Psychological consequences of false-positive screening mammograms in the UK. *BMJ Evidence-Based*. Medicine.

[7] Bond M, Garside R, Hyde C (2015). A crisis of visibility: the psychological consequences of false‐positive screening mammograms, an interview study. Br. J. Health Psychol..

[8] Lindberg LG, Svendsen M, Dømgaard M, Brodersen J (2013). Better safe than sorry: a long-term perspective on experiences with a false-positive screening mammography in Denmark. Health Risk Soc..

[9] Nelson HD, Tyne K, Naik A, Bougatsos C, Chan BK, Humphrey L (2009). Screening for breast cancer: an update for the US Preventive Services Task Force. Ann. Int. Med..

[10] Armstrong K, Moye E, Williams S, Berlin JA, Reynolds EE (2007). Screening mammography in women 40 to 49 years of age: a systematic review for the American College of Physicians. Ann. Int. Med..

[11] Brett J, Bankhead C, Henderson B, Watson E, Austoker J (2005). The psychological impact of mammographic screening. A systematic review. Psycho Oncol..

[12] Brewer NT, Salz T, Lillie SE (2007). Systematic review: the long-term effects of false-positive mammograms. Ann. Int. Med..

[13] Brett J, Austoker J (2001). Women who are recalled for further investigation for breast screening: psychological consequences 3 years after recall and factors affecting re‐attendance. J. Public Health.

[14] Cockburn J, Staples M, Hurley SF, De Luise T (1994). Psychological consequences of screening mammography. J. Med. Screen..

[15] Lampic C, Thurfjell E, Bergh J, Sjödén PO (2001). Short-and long-term anxiety and depression in women recalled after breast cancer screening. Euro. J. Cancer.

[16] Salz T, Richman AR, Brewer NT (2010). Meta‐analyses of the effect of false‐positive mammograms on generic and specific psychosocial outcomes. Psycho Oncol..

[17] Brett J, Austoker J, Ong G (1998). Do women who undergo further investigation for breast screening suffer adverse psychological consequences? A multi-centre follow-up study comparing different breast screening result groups five months after their last breast screening appointment. J. Public Health.

[18] Ong G, Austoker J, Brett J (1997). Breast screening: adverse psychological consequences one month after placing women on early recall because of a diagnostic uncertainty. A multicentre study. J. Med. Screen..

[19] Maxwell AJ, Beattie C, Lavelle J, Lyburn I, Sinnatamby R, Garnett S (2013). The effect of false positive breast screening examinations on subsequent attendance: retrospective cohort study. J. Med. Screen..

[20] Health Quality Ontario. (2016). Women’s experiences of inaccurate breast cancer screening results: a systematic review and qualitative meta-synthesis. Ont. Health Technol. Assess. Ser..

[21] Noyes J, Booth A, Flemming K, Garside R, Harden A, Lewin S (2018). Cochrane Qualitative and Implementation Methods Group guidance series—paper 3: methods for assessing methodological limitations, data extraction and synthesis, and confidence in synthesized qualitative findings. J. Clin. Epidemiol..

[22] Ring N., Ritchie K., Mandava L., Jepson R. A guide to synthesising qualitative research for researchers undertaking health technology assessments and systematic reviews. http://www.healthcareimprovementscotland.org/his/idoc.ashx?docid=04df262c-ec20-40e1-aef7-d2aa3ad03c06&version=-1 (2010).

[23] Tong A, Flemming K, McInnes E, Oliver S, Craig J (2012). Enhancing transparency in reporting the synthesis of qualitative research: ENTREQ. BMC Med. Res. Methodol..

[24] Noyes J, Booth A, Cargo M, Flemming K, Garside R, Hannes K (2018). Cochrane Qualitative and Implementation Methods Group guidance series—paper 1: introduction. J. Clin. Epidemiol..

[25] Moher D, Liberati A, Tetzlaff J, Altman DG (2009). Preferred reporting items for systematic reviews and meta-analyses: the PRISMA statement. Ann. Inter. Med..

[26] Williams, T., Shaw, R. in *International handbook of qualitative methods in sport and exercise*, pp 274–288 (eds B. Smith & A. C. Sparkes) (Routledge, London, 2016).

[27] Critical Appraisal Skills Programme (CASP) Qualitative Research Checklist. 10 questions to help you make sense of qualitative research. http://docs.wixstatic.com/ugd/dded87_25658615020e427da194a325e7773d42.pdf (2017).

[28] Levitt HM, Bamberg M, Creswell JW, Frost DM, Josselson R, Suárez-Orozco C (2018). Journal article reporting standards for qualitative primary, qualitative meta-analytic, and mixed methods research in psychology: the APA Publications and Communications Board task force report. Am. Psychol..

[29] Thomas J, Harden A (2008). Methods for the thematic synthesis of qualitative research in systematic reviews. BMC Med. Res. Methodol..

[30] Johnson RB (2017). Dialectical pluralism: a metaparadigm whose time has come. J. Mixed Methods Res..

[31] Bond M, Garside R, Hyde C (2015). Improving screening recall services for women with false-positive mammograms: a comparison of qualitative evidence with UK guidelines. BMJ Open.

[32] Solbjør M, Forsmo S, Skolbekken JA, Sætnan AR (2011). Experiences of recall after mammography screening—a qualitative study. Health Care Women Int..

[33] Fielding R, Lam TH (2007). The impact of a false-positive result from breast cancer mammography: a qualitative pilot study. Hong Kong Med. J..

[34] Padgett DK, Yedidia MJ, Kerner J, Mandelblatt J (2001). The emotional consequences of false positive mammography: African-American women’s reactions in their own words. Women Health.

[35] Thomson MD, Siminoff LA (2015). Perspectives on mammography after receipt of secondary screening owing to a false positive. Women’s Health Issues.

[36] Brodersen J, Thorsen H (2008). Consequences of Screening in Breast Cancer (COS-BC): development of a questionnaire. Scand. J. Prim. Health Care.

[37] Public Health England. Clinical nurse specialists in breast screening. https://www.gov.uk/government/publications/breast-screening-guidance-for-clinical-nurse-specialists/clinical-nurse-specialists-in-breast-screening (2019).

[38] Public Health England. Breast screening: clinical guidelines for screening assessment. www.gov.uk/government/publications/breast-screening-clinical-guidelines-for-screening-management (2016).

[39] NHS Digital. Breast Screening Programme. https://files.digital.nhs.uk/60/77DCCC/breast-screening-programme-eng-2017-18-report.pdf (2019).

[40] Kaushal A, Ramirez AJ, Warburton F, Forster AS, Linsell L, Burgess C (2017). “Promoting Early Presentation” intervention sustains increased breast cancer awareness in older women for three years: a randomized controlled trial. J. Med. Screen..

[41] Department of Health. *Improving Outcomes: A Strategy for Cancer*. (Department of Health, London, 2011).

[42] Forbes LJ, Ramirez AJ (2014). Expert group on Information about Breast Screening. Offering informed choice about breast screening. J. Med. Screen..

[43] Reyna VF (2008). A theory of medical decision making and health: fuzzy trace theory. Med. Decis. Making.

