# Correction: How do women experience a false-positive test result from breast screening? A systematic review and thematic synthesis of qualitative studies

**DOI:** 10.1038/s41416-021-01503-w

**Published:** 2021-07-30

**Authors:** Hannah Long, Joanna M. Brooks, Michelle Harvie, Anthony Maxwell, David P. French

**Affiliations:** 1 Manchester Centre for Health Psychology, School of Health Sciences, University of Manchester, Manchester, M13 9PL UK; 2 Nightingale Centre, Manchester University NHS Foundation Trust, Manchester, M23 9LT UK; 3 Division of Informatics, Imaging & Data Sciences, School of Health Sciences, University of Manchester, Manchester, M13 9PT UK

**Keywords:** Cancer screening, Human behaviour, Psychology

Correction to: *British Journal of Cancer* 10.1038/s41416-019-0524-4, published online 23 July 2019

The original version of this article unfortunately contained a mistake. The supplementary files are inaccessible. We sincerely apologize for the error. The original article has been corrected.

