# Supplementary material for: How do women experience a false-positive test result from breast screening? A systematic review and thematic synthesis of qualitative studies
Source: Br J Cancer. 2019 Jul 23;121(4):351–8. doi: 10.1038/s41416-019-0524-4 (PMC6738040; doi:10.1038/s41416-019-0524-4)
Supplement: Supplementary file 1 — Supplementary materials, tables, figures, and legends [file 41416_2019_524_MOESM1_ESM.docx]

**Supplementary materials 1. PRISMA Checklist**

| **Section/topic** | **#** | **Checklist item** | **Reported on page #** |
| --- | --- | --- | --- |
| **TITLE** | | | |
| Title | 1 | Identify the report as a systematic review, meta-analysis, or both. | 1,2,5 |
| **ABSTRACT** | | | |
| Structured summary | 2 | Provide a structured summary including, as applicable: background; objectives; data sources; study eligibility criteria, participants, and interventions; study appraisal and synthesis methods; results; limitations; conclusions and implications of key findings; systematic review registration number. |  |
| **INTRODUCTION** | | | |
| Rationale | 3 | Describe the rationale for the review in the context of what is already known. | 3-4 |
| Objectives | 4 | Provide an explicit statement of questions being addressed with reference to participants, interventions, comparisons, outcomes, and study design (PICOS). | 4 |
| **METHODS** | | | |
| Protocol and registration | 5 | Indicate if a review protocol exists, if and where it can be accessed (e.g., Web address), and, if available, provide registration information including registration number. | 5 |
| Eligibility criteria | 6 | Specify study characteristics (e.g., PICOS, length of follow-up) and report characteristics (e.g., years considered, language, publication status) used as criteria for eligibility, giving rationale. | 5  Table 1 |
| Information sources | 7 | Describe all information sources (e.g., databases with dates of coverage, contact with study authors to identify additional studies) in the search and date last searched. | 5 |
| Search | 8 | Present full electronic search strategy for at least one database, including any limits used, such that it could be repeated. | Appendix A |
| Study selection | 9 | State the process for selecting studies (i.e., screening, eligibility, included in systematic review, and, if applicable, included in the meta-analysis). | 5 |
| Data collection process | 10 | Describe method of data extraction from reports (e.g., piloted forms, independently, in duplicate) and any processes for obtaining and confirming data from investigators. | 6 |
| Data items | 11 | List and define all variables for which data were sought (e.g., PICOS, funding sources) and any assumptions and simplifications made. | 6 |
| Risk of bias in individual studies | 12 | Describe methods used for assessing risk of bias of individual studies (including specification of whether this was done at the study or outcome level), and how this information is to be used in any data synthesis. | 5-6 |
| Summary measures | 13 | State the principal summary measures (e.g., risk ratio, difference in means). | n/a |
| Synthesis of results | 14 | Describe the methods of handling data and combining results of studies, if done, including measures of consistency (e.g., I^2^) for each meta-analysis. | 6-7 |
| Risk of bias across studies | 15 | Specify any assessment of risk of bias that may affect the cumulative evidence (e.g., publication bias, selective reporting within studies). | n/a |
| Additional analyses | 16 | Describe methods of additional analyses (e.g., sensitivity or subgroup analyses, meta-regression), if done, indicating which were pre-specified. | n/a |
| **RESULTS** | | | |
| Study selection | 17 | Give numbers of studies screened, assessed for eligibility, and included in the review, with reasons for exclusions at each stage, ideally with a flow diagram. | 7  Figure 1 |
| Study characteristics | 18 | For each study, present characteristics for which data were extracted (e.g., study size, PICOS, follow-up period) and provide the citations. | 7-8 |
| Risk of bias within studies | 19 | Present data on risk of bias of each study and, if available, any outcome level assessment (see item 12). | 8  Appendix D |
| Results of individual studies | 20 | For all outcomes considered (benefits or harms), present, for each study: (a) simple summary data for each intervention group (b) effect estimates and confidence intervals, ideally with a forest plot. | 7-8 |
| Synthesis of results | 21 | Present results of each meta-analysis done, including confidence intervals and measures of consistency. | 8-14 |
| Risk of bias across studies | 22 | Present results of any assessment of risk of bias across studies (see Item 15). | n/a |
| Additional analysis | 23 | Give results of additional analyses, if done (e.g., sensitivity or subgroup analyses, meta-regression [see Item 16]). | n/a |
| **DISCUSSION** | | | |
| Summary of evidence | 24 | Summarize the main findings including the strength of evidence for each main outcome; consider their relevance to key groups (e.g., healthcare providers, users, and policy makers). | 14 |
| Limitations | 25 | Discuss limitations at study and outcome level (e.g., risk of bias), and at review-level (e.g., incomplete retrieval of identified research, reporting bias). | 16 |
| Conclusions | 26 | Provide a general interpretation of the results in the context of other evidence, and implications for future research. | 16-18 |
| **FUNDING** | | | |
| Funding | 27 | Describe sources of funding for the systematic review and other support (e.g., supply of data); role of funders for the systematic review. | 18 |

*From:*  Moher D, Liberati A, Tetzlaff J, Altman DG, The PRISMA Group (2009). Preferred Reporting Items for Systematic Reviews and Meta-Analyses: The PRISMA Statement. PLoS Med 6(7): e1000097. doi:10.1371/journal.pmed1000097

For more information, visit: **www.prisma-statement.org**.

**Supplementary materials 2**

ENTREQ checklist (Enhancing transparency in reporting the synthesis of qualitative research) *

| No. Item | Guide questions/description | Reported |
| --- | --- | --- |
|  |  | on Page # |
| 1. Aim | State the research question the synthesis addresses | 2,4 |
|  |  |  |
| 2. Synthesis | Identify the synthesis methodology or theoretical framework which underpins |  |
| methodology | the synthesis, and describe the rationale for choice of methodology (e.g. |  |
|  | meta-ethnography, thematic synthesis, critical interpretive synthesis, | 6-7 |
|  | grounded theory synthesis, realist synthesis, meta-aggregation, meta-study, |  |
|  | framework synthesis) |  |
|  |  |  |
| 3. Approach to | Indicate whether the search was pre-planned (comprehensive search |  |
| searching | strategies to seek all available studies) or iterative (to seek all available | 5 |
|  | concepts until they theoretical saturation is achieved) |  |
|  |  |  |
| 4. Inclusion criteria | Specify the inclusion/exclusion criteria (e.g. in terms of population, language, |  |
|  | year limits, type of publication, study type) | 5, Table 1 |
|  |  |  |
| 5. Data sources | Describe the information sources used (e.g. electronic databases (MEDLINE, |  |
|  | EMBASE, CINAHL, psycINFO), grey literature databases (digital thesis, policy |  |
|  | reports), relevant organisational websites, experts, information specialists, | 5 |
|  | generic web searches (Google Scholar) hand searching, reference lists) and |  |
|  | when the searches conducted; provide the rationale for using the data sources |  |
|  |  |  |
| 6. Electronic Search | Describe the literature search (e.g. provide electronic search strategies with |  |
| strategy | population terms, clinical or health topic terms, experiential or social | 5  Appendix C |
|  | phenomena related terms, filters for qualitative research, and search limits) |  |
|  |  |  |
| 7. Study screening | Describe the process of study screening and sifting (e.g. title, abstract and full |  |
| methods | text review, number of independent reviewers who screened studies) | 5,  Figure 1 |
|  |  |  |
| 8. Study | Present the characteristics of the included studies (e.g. year of publication, |  |
| characteristics | country, population, number of participants, data collection, methodology, | 7-8 |
|  | analysis, research questions) |  |
|  |  |  |
| 9. Study selection | Identify the number of studies screened and provide reasons for study |  |
| results | exclusion (e,g, for comprehensive searching, provide numbers of studies |  |
|  | screened and reasons for exclusion indicated in a figure/flowchart; for |  |
|  | iterative searching describe reasons for study exclusion and inclusion based on | 7  Figure 1 |
|  | modifications to the research question and/or contribution to theory |  |
|  | development) |  |
|  |  |  |
| 10. Rationale for | Describe the rationale and approach used to appraise the included studies or |  |
| appraisal | selected findings (e.g. assessment of conduct (validity and robustness), |  |
|  | assessment of reporting (transparency), assessment of content and utility of | 5-6 |
|  | the findings) |  |
|  |  |  |
| 11. Appraisal items | State the tools, frameworks and criteria used to appraise the studies or |  |
|  | selected findings (e.g. Existing tools: CASP, QARI, COREQ, Mays and Pope [25]; |  |
|  | reviewer developed tools; describe the domains assessed: research team, | 5-6 |
|  | study design, data analysis and interpretations, reporting) |  |
|  |  |  |
| 12. Appraisal | Indicate whether the appraisal was conducted independently by more than |  |
| process | one reviewer and if consensus was required | 6 |
|  |  |  |
| 13. Appraisal results | Present results of the quality assessment and indicate which articles, if any, |  |
|  | were weighted/excluded based on the assessment and give the rationale | 5-7  Appendix D |
|  |  |  |
| 14. Data extraction | Indicate which sections of the primary studies were analysed and how were |  |
|  | the data extracted from the primary studies? (e.g. all text under the headings  “results /conclusions” were extracted electronically and entered into a | 6 |
|  | computer software) |  |
|  |  |  |
| 15. Software | State the computer software used, if any | 5 |
|  |  |  |
| 16. Number of | Identify who was involved in coding and analysis | 6-7 |
| reviewers |  |  |
|  |  |  |
| 17. Coding | Describe the process for coding of data (e.g. line by line coding to search for | 6-7 |
|  | concepts) |  |
|  |  |  |
| 18. Study | Describe how were comparisons made within and across studies (e.g. |  |
| comparison | subsequent studies were coded into pre-existing concepts, and new concepts | 6-7 |
|  | were created when deemed necessary) |  |
|  |  |  |
| 19. Derivation of | Explain whether the process of deriving the themes or constructs was |  |
| themes | inductive or deductive | 6 |
|  |  |  |
| 20. Quotations | Provide quotations from the primary studies to illustrate themes/constructs, |  |
|  | and identify whether the quotations were participant quotations of the | 8-14 |
|  | author’s interpretation |  |
|  |  |  |
| 21. Synthesis | Present rich, compelling and useful results that go beyond a summary of the |  |
| output | primary studies (e.g. new interpretation, models of evidence, conceptual | 9-14 |
|  | models, analytical framework, development of a new theory or construct) |  |
|  |  |  |

- Reference: Tong A, Flemming K, McInnes E, Oliver SA, Craig J. Enhancing transparency in reporting the synthesis of qualitative research: ENTREQ. BMC Medical Research Methodology 2012, 12:181.

**Supplementary materials 3**

Search strategy and search results

Table 1. Search terms

| **Context** | ***Experiences, views, beliefs*** |
| --- | --- |
| **How** | qualitative* OR mixed method* OR thematic analysis OR thematically OR theme* OR grounded theory OR grounded analysis OR ethnograph* OR content analysis OR discourse analysis OR narrative analysis OR conversation analysis OR hermeneutic* OR phenomenology OR interview* OR focus group* OR view* OR experienc* OR opinion* OR attitude* OR perce* OR belie* OR feel* OR understand* OR perspective*  AND |
| **Issue** | false*positive* OR abnormal OR diagnostic uncertaint* OR psychological impact OR psychological consequence* OR psychological factor* OR psychological effect* OR recall* OR further investigation  AND |
| **Population** | mammogra* OR breast screen* OR breast scan* |

Reference for CHIP: Williams & Shaw, 2016

Table 2. EMBASE search results (run on 23^rd^ January 2018)

|  | Search term | Number of results |
| --- | --- | --- |
| 1 | qualitative research/ or qualitative analysis/ or qualitative*.mp. | 286490 |
| 2 | mixed method*.mp. | 14813 |
| 3 | thematic analysis.mp. or thematic analysis/ | 14447 |
| 4 | thematically.mp. | 4715 |
| 5 | theme*.mp. | 83191 |
| 6 | grounded theory.mp. or grounded theory/ | 11267 |
| 7 | ethnography/ or ethnograph*.mp. | 10098 |
| 8 | content analysis.mp. or content analysis/ | 24583 |
| 9 | discourse analysis.mp. or discourse analysis/ | 2095 |
| 10 | narrative analysis.mp. | 1014 |
| 11 | conversation analysis.mp. | 608 |
| 12 | hermeneutics/ or hermeneutic*.mp. | 3424 |
| 13 | phenomenology.mp. or phenomenology/ | 14832 |
| 14 | interview*.mp. or interview/ | 404675 |
| 15 | focus group*.mp. | 42703 |
| 16 | attitude*.mp. or attitude/ | 459947 |
| 17 | view*.mp. | 514823 |
| 18 | experienc*.mp. | 1297978 |
| 19 | opinion*.mp. | 127598 |
| 20 | perce*.mp. | 1383982 |
| 21 | belie*.mp. | 321460 |
| 22 | feel*.mp. | 111921 |
| 23 | understand*.mp. | 1109389 |
| 24 | perspective*.mp. | 307160 |
| 25 | 1 or 2 or 3 or 4 or 5 or 6 or 7 or 8 or 9 or 10 or 11 or 12 or 13 or 14 or 15 or 16 or 17 or 18 or 19 or 20 or 21 or 22 or 23 or 24 | 5000756 |
| 26 | false positive result/ or false*positive*.mp. | 22099 |
| 27 | abnormal.mp. | 428378 |
| 28 | diagnostic uncertaint*.mp. | 1750 |
| 29 | psychological impact.mp. | 3826 |
| 30 | psychological consequence*.mp. | 2495 |
| 31 | psychological factor*.mp. | 12083 |
| 32 | psychological effect*.mp. | 4368 |
| 33 | recall*.mp. | 91093 |
| 34 | further investigation.mp. | 93777 |
| 35 | 26 or 27 or 28 or 29 or 30 or 31 or 32 or 33 or 34 | 652726 |
| 36 | cancer screening/ or mammography/ or mammogra*.mp. | 107094 |
| 37 | breast screen*.mp. | 2863 |
| 38 | breast scan*.mp. | 131 |
| 39 | 36 or 37 or 38 | 107756 |
| 40 | 25 and 35 and 39 | 2572 |
| 41 | limit 40 to yr=”1970-Current” | 2568 |

Table 3. MEDLINE search results (run on 23^rd^ January, 2018)

|  | Search term | Number of results |
| --- | --- | --- |
| 1 | QUALITATIVE RESEARCH/ or qualitative*.mp. | 189891 |
| 2 | mixed method*.mp. | 8919 |
| 3 | thematic analysis.mp. | 7835 |
| 4 | thematically.mp. | 2858 |
| 5 | theme*.mp. | 52817 |
| 6 | grounded theory.mp. or Grounded Theory/ | 7741 |
| 7 | ethnograph*.mp. | 7290 |
| 8 | content analysis.mp. | 15886 |
| 9 | discourse analysis.mp. | 1191 |
| 10 | narrative analysis.mp. | 728 |
| 11 | conversation analysis.mp. | 384 |
| 12 | hermeneutic*.mp. or HERMENEUTICS/ | 2680 |
| 13 | phenomenology.mp. | 5894 |
| 14 | INTERVIEW/ or interview*.mp. | 288208 |
| 15 | Focus Groups/ or focus group*.mp. | 33365 |
| 16 | ATTITUDE/ or attitude*.mp. | 355388 |
| 17 | view*.mp. | 355268 |
| 18 | experienc*.mp. | 817899 |
| 19 | opinion*.mp. | 89195 |
| 20 | perce*.mp. | 1013834 |
| 21 | belie*.mp. | 220538 |
| 22 | feel*.mp. | 66821 |
| 23 | understand*.mp. | 753824 |
| 24 | perspective*.mp. | 208184 |
| 25 | 1 or 2 or 3 or 4 or 5 or 6 or 7 or 8 or 9 or 10 or 11 or 12 or 13 or 14 or 15 or 16 or 17 or 18 or 19 or 20 or 21 or 22 or 23 or 24 | 3443179 |
| 26 | False Positive Reactions/ or false*positive*.mp. | 26315 |
| 27 | abnormal.mp. | 285988 |
| 28 | diagnostic uncertaint*.mp. | 956 |
| 29 | psychological impact.mp. | 2201 |
| 30 | psychological consequence*.mp. | 1592 |
| 31 | psychological factor*.mp. | 12783 |
| 32 | psychological effect*.mp. | 2970 |
| 33 | recall*.mp. | 70505 |
| 34 | further investigation.mp. | 55147 |
| 35 | 26 or 27 or 28 or 29 or 30 or 31 or 32 or 33 or 34 | 453531 |
| 36 | Mammography/ or mammogra*.mp. | 34197 |
| 37 | breast screen*.mp. | 1887 |
| 38 | breast scan*.mp. | 86 |
| 39 | 36 or 37 or 38 | 34854 |
| 40 | 25 and 35 and 39 | 1020 |
| 41 | limit 40 to yr=”1970-Current” | 1020 |

Table 4. CINAHL search results (run on 23^rd^ January, 2018)

| (MH "Qualitative Studies") OR "qualitative*" OR (MH "Grounded Theory") OR (MH "Content Analysis") OR "thematic analysis" OR "narrative analysis" OR "conversation analysis" OR "interview*" OR "focus group*" OR "view*" OR "experienc*" OR "opinion*" OR "attitude*" OR "perce*" OR "belie*" OR "feel*" OR "understand*" OR "perspective*" |
| --- |
| AND |
| (MH "False Positive Results") OR "false positive*" OR "abnormal" OR "diagnostic uncertaint*" OR "psychological impact" OR "psychological consequence*" OR "psychological factor*" OR "psychological effect*" OR "recall*" OR "further investigation" |
| AND |
| (MH "Mammography") OR "mammogra*" OR (MH "Rescreening") OR "breast screen*" OR "breast scan" |
| Limited to 1970-current |
| 460 RESULTS |

Table 5. PsycINFO search results (run on 23^rd^ January, 2018)

|  | Search term | Number of results |
| --- | --- | --- |
| 1 | exp QUALITATIVE RESEARCH/ or qualitative*.mp. | 148742 |
| 2 | mixed method*.mp. | 18104 |
| 3 | thematic analysis.mp. | 8596 |
| 4 | thematically.mp. | 2803 |
| 5 | theme*.mp. | 99170 |
| 6 | grounded theory.mp. or exp Grounded Theory/ | 13706 |
| 7 | ethnograph*.mp. | 25676 |
| 8 | content analysis.mp. or exp Content Analysis/ | 30598 |
| 9 | discourse analysis.mp. or exp Discourse Analysis/ | 10970 |
| 10 | narrative analysis.mp. | 2054 |
| 11 | conversation analysis.mp. | 1854 |
| 12 | exp HERMENEUTICS/ or hermeneutic*.mp. | 6609 |
| 13 | phenomenology.mp. or exp PHENOMENOLOGY/ | 20579 |
| 14 | interview*.mp. | 351447 |
| 15 | focus group*.mp. | 30021 |
| 16 | attitude*.mp. | 397430 |
| 17 | view*.mp. | 285899 |
| 18 | experienc*.mp. | 617268 |
| 19 | opinion*.mp. | 51336 |
| 20 | perce*.mp. | 698039 |
| 21 | belie*.mp. | 196791 |
| 22 | feel*.mp. | 132986 |
| 23 | understand*.mp. | 457233 |
| 24 | perspective*.mp. | 255885 |
| 25 | 1 or 2 or 3 or 4 or 5 or 6 or 7 or 8 or 9 or 10 or 11 or 12 or 13 or 14 or 15 or 16 or 17 or 18 or 19 or 20 or 21 or 22 or 23 or 24 | 2232495 |
| 26 | false*positive*.mp. | 3680 |
| 27 | abnormal.mp. | 38770 |
| 28 | diagnostic uncertaint*.mp. | 203 |
| 29 | psychological impact.mp. | 2456 |
| 30 | exp Psychological Consequence/ psychological consequence*.mp. | 2683 |
| 31 | psychological factor*.mp. | 10672 |
| 32 | psychological effect*.mp. | 4617 |
| 33 | recall*.mp. | 59137 |
| 34 | further investigation.mp. | 9840 |
| 35 | 26 or 27 or 28 or 29 or 30 or 31 or 32 or 33 or 34 | 130211 |
| 36 | exp Cancer Screening/ or mammogra*.mp. | 5268 |
| 37 | breast screen*.mp. | 200 |
| 38 | breast scan*.mp. | 0 |
| 39 | 36 or 37 or 38 | 5310 |
| 40 | 25 and 35 and 39 | 319 |
| 41 | limit 40 to yr=”1970-Current” | 319 |

Table 6. Web of Science Core Collection search results (run on 5^th^ February, 2018)

|  |  |  |
| --- | --- | --- |
| 1 | TS=(qualitative* or mixed method* or thematic analysis or thematically or theme* or grounded theory or grounded analysis or ethnograph* or content analysis or discourse analysis or narrative analysis or conversation analysis or hermeneutic* or phenomenology or interview* or focus group* or view* or experienc* or opinion* or attitude* or perce* or belie* or feel* or understand* or perspective*)  *Indexes=SCI-EXPANDED, SSCI, A&HCI, CPCI-S, CPCI-SSH, BKCI-S, BKCI-SSH, ESCI, CCR-EXPANDED, IC Timespan=All years* | 7412452 |
| 2 | TS=(false positive* or abnormal or diagnostic uncertaint* or psychological impact or psychological consequence* or psychological factor* or psychological effect* or recall* or further investigation)  *Indexes=SCI-EXPANDED, SSCI, A&HCI, CPCI-S, CPCI-SSH, BKCI-S, BKCI-SSH, ESCI, CCR-EXPANDED, IC Timespan=All years* | 786498 |
| 3 | TS=(mammogram* or breast screen* or breast scan*)  *Indexes=SCI-EXPANDED, SSCI, A&HCI, CPCI-S, CPCI-SSH, BKCI-S, BKCI-SSH, ESCI, CCR-EXPANDED, IC Timespan=All years* | 75886 |
| 4 | #3 AND #2 AND #1  *Indexes=CPCI-S, CPCI-SSH, BKCI-S, BKCI-SSH Timespan=1970-2018* | 377 |

Table 7. ProQuest Dissertations and Theses Global search results (run on 6^th^ February, 2018)

| (qualitative* OR interview* OR "focus group*") AND ("false positive*") AND (mammogra* OR "breast screen*") |
| --- |
| Limited to January 1^st^ 1970 to December 31^st^ 2018 and ‘Doctoral dissertations’ |
| 1586 RESULTS |

Table 8. OpenGrey search results (run on 6^th^ February, 2018)

| false positive AND mammogra* |
| --- |
| 3 RESULTS |

NB. No limits possible.

Table 9. NHS Evidence search results (run on 6^th^ February, 2018)

| (false positive* OR abnormal) AND (mammogra* OR breast screen*) AND (qualitative* OR interview* OR focus group*) |
| --- |
| Limited to Primary Research and Secondary Evidence. |
| 544 RESULTS |

NB. No limit on ‘year of publication’ possible.

**Supplementary materials 4**

Summary and detailed results of the quality appraisal using the CASP

Summary of the quality appraisal results

| Authors (year) | CASP question | | | | | | | | | |
| --- | --- | --- | --- | --- | --- | --- | --- | --- | --- | --- |
|  | 1. Was there a clear statement of the aims of the research? | 2. Is a qualitative methodology appropriate? | 3. Was the research design appropriate to address the aims of the research? | 4. Was the recruitment strategy appropriate to the aims of the research? | 5. Was the data collected in a way that addressed the research issue? | 6. Has the relationship between researcher and participants been adequately considered? | 7. Have ethical issues been taken into consideration? | 8. Was the data analysis sufficiently rigorous? | 9. Is there a clear statement of findings? | 11. Are the study’s theoretical underpinnings clear, consistent and conceptually coherent? |
| Bolejko et al. (2014) | Yes | Yes | Yes | Somewhat | Yes | No | Somewhat | Somewhat | Yes | No |
| Bond et al. (2015a) | Yes | Yes | Yes | Yes | Yes | Yes | Somewhat | Somewhat | Yes | No |
| Bond et al. (2015b) | Yes | Yes | Yes | Can’t tell | Can’t tell | Somewhat | Somewhat | Can’t tell | Yes | No |
| Fielding & Lam (2007) | No | Can’t tell | Can’t tell | Can’t tell | Can’t tell | No | No | No | No | No |
| Lindberg et al. (2013) | Somewhat | Yes | Yes | No | Can’t tell | No | Somewhat | Yes | Somewhat | Somewhat |
| Padgett et al. (2001) | No | Can’t tell | Can’t tell | No | No | Yes | Somewhat | No | No | No |
| Solbjor et al. (2011) | Somewhat | Yes | Yes | Yes | Somewhat | Yes | Somewhat | No | No | No |
| Thomson et al. (2015) | Yes | Yes | Somewhat | Yes | Somewhat | No | Somewhat | No | Somewhat | Can’t tell |

NB. Questions 1 and 2 are screening questions.

Detailed responses of quality appraisal

Bolejko et al.^5^

| **1. Was there a clear statement of the aims of the research?** |
| --- |
| Yes |
| **2. Is a qualitative methodology appropriate?** |
| Yes |
| **3. Was the research design appropriate to address the aims of the research?** |
| Yes   - The authors have not explicitly justified why their chosen method is more suitable and appropriate than other methods, but the design is appropriate to address their research aims. |
| **4. Was the recruitment strategy appropriate to the aims of the research?** |
| Somewhat   - Purposive sampling was used to recruit a varied sample. The participant variables that were specially sought have been provided. - The authors specifically recruited women who showed evidence of short- and/or longer-term psychological consequences of having a false positive screening result (indicated by scores on the COS-BC questionnaire of psychological consequences). Arguably, this recruitment strategy is only partially appropriate to address the research aims as the authors sought out women who had a relatively negative experience. It may be the case that women who did not show evidence of psychological consequences in their COS-BC score did so because they have coped well with the situation and, given that the authors were looking to describe how women coped with their experience, it may have been worthwhile to recruit and interview such women (e.g. did they cope better? If so, how?). |
| **5. Was the data collected in a way that addressed the research issue?** |
| Yes   - Data were collected through semi-structured interviews, which were audio-recorded before being transcribed verbatim. - Participants were given choice over where the interview took place, although the locations of the interviews have not been reported. - The topic guide and flow of the interview has been described well. |
| **6. Has the relationship between researcher and participants been adequately considered?** |
| No   - There is no evidence that the researcher(s) examined their own role, potential bias or influence during formulation of the research question, recruitment or data collection and analysis. - In the Discussion section, the authors briefly mention the concept of ‘confirmability’ and provide a reference from 1985 in support. The authors suggest that the data should speak freely for itself without being impacted by the views of the researcher(s) – some qualitative researchers would argue that it is not possible to totally remove the views of the researchers from data analysis and reporting of findings. The authors state that by providing quotations from 10 of 13 transcripts, they have strengthened the confirmability of their findings, but this is somewhat unconvincing. - The authors state that it was essential that they were aware of their preunderstandings of the study context. This is reported presumably because the authors were needed to be considerate of their views before analysing data, so they can achieve ‘confirmability’, but how this was achieved in practice is unclear. |
| **7. Have ethical issues been taken into consideration?** |
| Somewhat   - The study was granted ethical approval, indicating that ethical issues were sufficiently considered before the study commenced. - However, certain key ethical processes have not been described (e.g. how contact information for potential participants was obtained, who was responsible for corresponding with potential participants, how the authors ensured consent was fully informed, and there is no mention of a participant information sheet, the protocol for data protection, and the process of anonymising interview data, although participant quotations reported in the Results have been anonymised). It is therefore not possible to tell whether ethical standards were maintained throughout the course of the study. |
| **8. Was the data analysis sufficiently rigorous?** |
| Somewhat   - There is a reasonably good, albeit jargon-heavy, description of the analysis process (some [not all] key definitions are reported and descriptions of process are included). Given that the description of the analysis is quite technical, definitions for, and an explanation of the methodological differences between, codes, categories, themes and how they are related would have been useful. - The bulk of the analysis was undertaken by two authors, who met to discuss their progress on three occasions. All authors agreed the final results. - In three of the four themes, the participant quotations provided to evidence the authors’ interpretation do not always support the wider narrative or theme name (the other theme is too short for this to be an issue, but the short theme is an issue in itself). - The narrative and supporting quotations for theme 3 is very brief compared with the other themes, suggesting there was a lack of data to support this theme. It perhaps should not have been a standalone theme. |
| **9. Is there a clear statement of findings?** |
| Yes   - There is a reasonably clear statement of the main findings and discussion for and against the authors’ interpretations of the data. - The authors have made an effort to consider methodological issues (e.g. credibility, transferability, dependability and confirmability of findings) related to their qualitative findings. |
| **10. How valuable is the research?** |
| There are clear sections in the Discussion dedicated to the relevance of their findings to clinical practice (describing the authors’ views on the importance of their findings) and a Box on the Title page labelled ‘What does this paper contribute to the wider global clinical community?’ (describing the value of the research). The authors have made some effort to identify avenues for future research, including implications, i.e. counselling interventions to support women experiencing adverse psychological consequences of screening. However, this suggestion may not be practical, affordable or implementable in healthcare systems. |
| **11. Are the study’s theoretical underpinnings (e.g. ontological and epistemological assumptions; guiding theoretical framework(s)) clear, consistent and conceptually coherent?** |
| No   - The study’s theoretical underpinnings have not been reported and are therefore unclear. - The authors reference using a data analysis procedure by Granehim and Lundman (2004). The referenced authors state that reality is interpreted in various ways, understanding depends on subjective interpretation, text always involves multiple meanings and interpretations, and that this knowledge is essential when discussing trustworthiness of findings. It is not clear whether the authors honoured this approach; indeed, the authors efforts to suggest the data should speak for itself and that it is important to remove the authors’ views from data analysis suggest that perhaps Granehim and Lundman’s approach was not followed. |

Bond et al.^7^

| **1. Was there a clear statement of the aims of the research?** |
| --- |
| Yes |
| **2. Is a qualitative methodology appropriate?** |
| Yes |
| **3. Was the research design appropriate to address the aims of the research?** |
| Yes   - The authors have explained that they were looking to understand the ‘meaning’ behind the experience of having a false positive breast screening result, to gain a deeper understanding of women’s experiences. |
| **4. Was the recruitment strategy appropriate to the aims of the research?** |
| Yes   - There is a reasonably good description of how participants were recruited. The recruitment strategy is explained and justified. |
| **5. Was the data collected in a way that addressed the research issue?** |
| Yes   - The authors have broadly justified why semi-structured interviews were approach for their research question. - The topic guide was based on a recent systematic review (Bond et al., 2013) and briefly but sufficiently described. The questions were piloted by both women who had and had not previously received a false positive breast screening result. - The authors reported that the interviewer determined that data saturation had been achieved, but what is meant by this or how it was realised is unclear. |
| **6. Has the relationship between researcher and participants been adequately considered?** |
| Yes   - The relationship between researcher and participants has been described in the Methods and in Table 2. Specifically, the authors have described the interviewer’s research background and experience, and how this influenced the way that she approached the interviews. The authors have also described the interviewer’s demeanour and her awareness of both verbal and non-verbal cues, and how this may have affected disclosure of information by participants. - Piloting the interview questions with women who had and had not previously received a false positive breast screening result was a nice consideration and could have provided valuable insight into the potentially more influential position of the interviewer. - The authors have described that the researcher respected what the participants were saying; taking their statements at face value and not second-guessing the participants’ motives for sharing particular pieces of information. |
| **7. Have ethical issues been taken into consideration?** |
| Somewhat   - The study was granted ethical approval, indicating that ethical issues were sufficiently considered before the study commenced. - However, certain key ethical processes have not been described (e.g. no mention of obtaining informed consent from participants, giving participants study documents including a participant information sheet, the protocol for data protection, who corresponded with potential participants during recruitment, etc.). It is therefore not possible to tell whether the ethical practices for which they received approval were adhered to throughout the course of the study. |
| **8. Was the data analysis sufficiently rigorous?** |
| Somewhat   - The authors’ use of the data analysis method interpretative phenomenological analysis (IPA) (gaining understanding of experience as it is lived, through an in-depth individualistic analysis) with a larger-than-is-typical-for-IPA sample has been fairly well justified, although it remains a little unclear how the authors’ version of IPA (one which moves from individual-level analysis to an analysis across the group of transcripts, to identify common themes, and how these are inter-related, from the shared experiences of a group) is distinct from thematic analysis. - The majority of the themes map directly onto the topic guide, insomuch as the themes are presented chronologically through the diagnostic journey, much like the flow of the topic guide. We are unsure whether this is a result of themes developed inductively through coding of the data (as the authors suggest). The final theme (‘Reflections’) stands apart from the chronological themes, but, arguably, its narrative could have been worked into the rest of the analysis, but was considered separate by the authors. These issues may bring into question the suitability of IPA as an analysis method for larger samples. - The process of selecting participant quotations for use in the final narrative is described well. Negative cases have been presented well. |
| **9. Is there a clear statement of findings?** |
| Yes   - There is a reasonably good summary of the main findings. - The authors have clearly considered methodological issues associated with their qualitative findings (e.g. validation of findings, Yardley’s principles of sensitivity to context, commitment and rigour, transparency and coherency of findings). |
| **10. How valuable is the research?** |
| The authors discuss the study’s contribution to existing academic knowledge, but they have not made any recommendations for future research or practice. Doing so would have strengthened the value of this research. The authors did not mention their other paper published from this dataset – doing so may have added valuable or at least interesting insight beyond that of this paper on its own. |
| **11. Are the study’s theoretical underpinnings (e.g. ontological and epistemological assumptions; guiding theoretical framework(s)) clear, consistent and conceptually coherent?** |
| No   - The study’s theoretical underpinnings have not been reported and are therefore unclear. - There may be some confusion regarding the implicit guiding theoretical frameworks due to the issues regarding the suitability of IPA for this study. |

Bond et al.^29^

| **1. Was there a clear statement of the aims of the research?** |
| --- |
| Yes |
| **2. Is a qualitative methodology appropriate?** |
| Yes |
| **3. Was the research design appropriate to address the aims of the research?** |
| Yes   - The authors have justified the use of a qualitative design. - A survey design may have been equally appropriate, and may have broadened the possible geographic scope for the study (which is relevant to the authors’ aim of comparing current practice with the NHS breast screening programme guidelines for the information needs of women recalled following mammography screening). |
| **4. Was the recruitment strategy appropriate to the aims of the research?** |
| Can’t tell   - The explanation of the recruitment strategy is somewhat brief and lacking in detail. - A relatively large amount of word space is given over to explain the UK Index of Multiple Deprivation, which was used to purposively sample participants. The authors reported that participants’ characteristic were found to fulfil the criteria of diversity and that detailed information about educational level, income and social group would aid interpretation of the results, but this is simply a statement and the authors do not offer their own interpretations of these factors in light of the findings, despite being the best placed to do so. The authors later report, in the Discussion, that there was no link between participants’ demographic characteristics and the issues they raised. The CASP hints that authors should explain why the selected participants were the most appropriate to provide access to the type of knowledge sought by the study – the authors could have done this better. - Referencing their sister paper (Bond et al., 2015a) would have resolved some of the issues related to the scarceness of detail regarding participant recruitment, as it is reported in greater detail in this paper. |
| **5. Was the data collected in a way that addressed the research issue?** |
| Can’t tell   - Description of data collection is generally quite thin. - The authors report that the topic guide was based on the results of the latest systematic review (Bond et al., 2013), but no further detail is given. As the reader, it is not easy to ascertain how the results of the systematic review of quantitative studies have influenced the topic guide of this qualitative study. Further, although it is not stated by the authors, it would appear that the interviews conducted and data collected for Bond et al. (2015a) and Bond et al. (2015b) are the same. It would have been useful and interesting to learn how the two research questions were handled within one topic guide. |
| **6. Has the relationship between researcher and participants been adequately considered?** |
| Somewhat   - There is no evidence that the researchers examined their role, bias and influence in the development of the research questions. However, there is some good consideration of the relationship between researcher and participants in relation to data collection and data analysis, but these are delivered as general statements of practice; often the ‘how’ and ‘why’ is not addressed. - There is a nice consideration of the analyst’s lack of first-hand experience of breast screening. |
| **7. Have ethical issues been taken into consideration?** |
| Somewhat   - The study was granted ethical approval, indicating that ethical issues were sufficiently considered before the study commenced. - However, certain key ethical processes have not been described (e.g. how contact information for potential participants was obtained, who was responsible for corresponding with potential participants, how the authors ensured consent was fully informed, and there is no mention of a participant information sheet, the protocol for data protection). It is therefore not possible to tell whether the ethical practices for which they received approval were adhered to throughout the course of the study. |
| **8. Was the data analysis sufficiently rigorous?** |
| Can’t tell   - The authors used inductive qualitative content analysis, but the description is brief. - It is not clear what process the authors followed to compare their analysis findings with the existing UK guidelines for the NHS breast screening programme. It appears that the authors developed their own list of screening service suggestions, from their analysis findings, and compared these with the guidelines (Table 3). This procedure is not reported in the methods. - The Results is written clearly, but parts of the narrative feel thin. Despite this, a good number of participant quotations are used to evidence the authors’ interpretations. |
| **9. Is there a clear statement of findings?** |
| Yes   - The authors summarise their findings nicely, linking them back to their original research aims. - The authors have validated their findings using Yardley’s principles for quality in qualitative research. |
| **10. How valuable is the research?** |
| The authors compared their findings to the NHS breast screening programme guidelines and suggest how aspects of these guidelines could be upheld better in future, and this is a valuable and original contribution. The findings are related back to existing literature and grounded in what is already known. The authors report pragmatic practice implications of their findings, and identify avenues for future research.  It may be the case that the reporting standards were compromised by a limited journal word count. |
| **11. Are the study’s theoretical underpinnings (e.g. ontological and epistemological assumptions; guiding theoretical framework(s)) clear, consistent and conceptually coherent?** |
| No   - The study’s theoretical underpinnings have not been reported and are therefore unclear. |

Fielding & Lam^31^

| **1. Was there a clear statement of the aims of the research?** |
| --- |
| No |
| **2. Is a qualitative methodology appropriate?** |
| Can’t tell   - As the research aims were not clearly stated, it is difficult to say whether a qualitative methodology was totally appropriate. However, given what they have done and reported, it does not appear to be inappropriate. |
| **3. Was the research design appropriate to address the aims of the research?** |
| Can’t tell   - Similarly to above, as the research aims were not clearly stated, it is not possible to confidently say whether a qualitative interview design was totally appropriate, but again, it is not inappropriate. |
| **4. Was the recruitment strategy appropriate to the aims of the research?** |
| Can’t tell   - It is not possible to tell whether the recruitment strategy was appropriate for the study because the authors have not reported clear research aims. - The authors reported that they managed to isolate the false positive experience, in the group of women they recruited; however, they specifically targeted recruitment at women who had had a false positive breast screening result diagnosed by fine needle aspiration (FNA) biopsy (rather than other types of follow up assessment e.g. repeat mammogram, ultrasound). Other research suggests FNA is more distressing than other types of follow up assessment and thus this recruitment decision may have influenced the type of interview data collected (e.g. biased it towards more distressing experiences). This issue may indicate a difference in the standard diagnostic procedures of Western screening service programmes versus those in Hong Kong. The authors state that, in Hong Kong, women “generally undergo a fine needle aspiration biopsy” following an abnormal mammography; this statement implies that other follow up assessments are also used, perhaps in place of FNA. |
| **5. Was the data collected in a way that addressed the research issue?** |
| Can’t tell   - There is a lack of sufficient detail in the methods regarding data collection. |
| **6. Has the relationship between researcher and participants been adequately considered?** |
| No   - There is a lack of consideration for the relationship between researcher and participants has been considered. It would have been particularly important to do so in this study, as the authors report that a public debate took place in Hong Kong over the risks and benefits of breast screening, during the time of the study, and that access to potential participants was withdrawn because one of the researchers was perceived as opposing breast screening. The authors did not critically examine whether the authors personal beliefs may or may not have biased the interviews, influenced the relationship with the participants, or the research design and data analysis. |
| **7. Have ethical issues been taken into consideration?** |
| No   - There is no evidence that of ethical approval for this study or that ethical issues were sufficiently considered. For example, the authors report obtaining informed consent from participants, but it is not clear how this was achieved, and there is no mention of providing potential participants with study documents, or of the procedure for data protection. |
| **8. Was the data analysis sufficiently rigorous?** |
| No   - The authors report taking a phenomenological approach, but it is not clear what is meant by this or what it entailed. The description of the data analysis procedure is overly complex and jargon-heavy. As the reader, it is difficult to understand what the analysts did, and it would not be possible to replicate the steps of the analysis based on the authors’ description. No references were provided. - The spread of supporting data excerpts from participants is not equal (which is arguably at odds with phenomenological approaches to data analysis). Often, the data (participant quotations) is left to speak for itself and the supporting narrative is descriptively thin. - The themes feel unfinished; there is narrative overlap between some themes (e.g. the theme ‘experiences of false positive labelling’ and the theme ‘results of the fine needle aspiration’ cover similar ground). Often, the narrative does not support its associated, overarching theme and, as the reader, it feels that no clear themes come through from the narrative and data presented. |
| **9. Is there a clear statement of findings?** |
| No   - There is no clear statement of findings. The few conclusions that have been reported feel at odds with the results. For example, the opening sentence to the Discussion states that, overall, the women report a positive experience, but this has not come across in the findings. - There is a lack of discussion for and against the authors’ interpretations and the credibility of their findings. - The authors do not try to relate their findings back to an (otherwise unreported) overall research aim. |
| **10. How valuable is the research?** |
| This pilot study is of poor quality. Furthermore, the authors explicitly chose not to make any practice or research recommendations based on their findings because they believed that their sample size was too small and data saturation was unlikely. The results and conclusions should be interpreted with appropriate caution. |
| **11. Are the study’s theoretical underpinnings (e.g. ontological and epistemological assumptions; guiding theoretical framework(s)) clear, consistent and conceptually coherent?** |
| No   - The study’s theoretical underpinnings have not been reported and are therefore unclear. - What is more, the authors report taking a phenomenological analytic approach to data analysis, which implies that they may have analysed their data with an idiographic focus on the subjective experiences of their participants; however, there is no evidence that this was taken into consideration in the description of the data analysis or the reporting of findings. |

Lindberg et al.^8^

| **1. Was there a clear statement of the aims of the research?** |
| --- |
| Somewhat |
| **2. Is a qualitative methodology appropriate?** |
| Yes |
| **3. Was the research design appropriate to address the aims of the research?** |
| Yes   - While the research aims are a little unclear, the authors have provided reasonable justification for using a qualitative interview design. |
| **4. Was the recruitment strategy appropriate to the aims of the research?** |
| No   - The authors specifically sampled women who had been the most negatively affected by having a false positive breast screening result. The authors justified this on the basis that (i) research in the area of false positive breast screening results was limited and (ii) women who have been more greatly affected will have greater insight. Arguably, this was not an appropriate or justified recruitment strategy to address the aims of the research, which were more general and did not specify the ‘most affected women’. Being more greatly affected by something does not necessarily lead to greater insight. - The authors approached women with the most negative scores on a questionnaire of the psychological consequences of having a false positive breast screening to be participants. The authors reported that, after taking questionnaire scores into account, they aimed for variation in other sociodemographic characteristics (e.g. marital status, age). However, invitations to participate in the study were sent to 23 women who had the most negative scores on the psychological consequences questioning – suggesting that negative scores were prioritised over diversity in other characteristics. |
| **5. Was the data collected in a way that addressed the research issue?** |
| Can’t tell   - It is not possible to tell from the information reported in the methods whether the data were collected in a way that addressed the research issue. For example, it is not clear whether interviews were structured or unstructured or whether closed or open questions were used. There is no information about the topic guide or the flow/direction the interviews took. |
| **6. Has the relationship between researcher and participants been adequately considered?** |
| No   - There is no evidence that the relationship between the researcher and participants has been considered. The authors seemed to follow Strauss and Corbin’s method of grounded theory; this approach encourages researchers to be personally and actively engaged with the research in order to describe and understand participants’ perceptions. |
| **7. Have ethical issues been taken into consideration?** |
| Somewhat   - In Denmark, ethical approval for qualitative studies is not required. However, the authors have taken certain steps to ensure some ethical standards were met – there is evidence of obtaining informed consent from participants before they were interviewed; the use of pseudonyms in the transcripts to protect participants’ identities; the interviews were conducted in the participants’ homes. |
| **8. Was the data analysis sufficiently rigorous?** |
| Yes   - The transcripts were double coded by two authors, who met to discuss and agree on their codes. The analysis appeared to be thoroughly iterative. - The analysis process has been described in reasonably good detail. However, the authors report that after they had finished their analysis, they elaborated and externally validated their findings by applying existing theory in a further analysis. The process of analysis had been described well up to this point, but this phase of analysis needed to be explained and justified. As a result, participants’ experiences and the accompanying narrative is often interpreted and explained using existing theory; alternative explanations, closer to the data, were possible but not always sought, and this may have been a chance to stay closer to the participants’ experiences and data and to build (novel) theory. |
| **9. Is there a clear statement of findings?** |
| Somewhat   - There is a discussion of the findings but it is not explicit. The structure of the Discussion probably reflects differences between disciplines in academic report writing, which may also explain the absence of practice and research recommendations and consideration of the credibility of their findings. |
| **10. How valuable is the research?** |
| The authors report an interesting narrative and go to great lengths to explain their findings using existing theory. In this sense, it is grounded nicely in relevant theory. However, they do not consider their findings in relation to current health care practice or policy. They do not make any practice or research recommendations. They do not consider the strengths and limitations of their study. |
| **11. Are the study’s theoretical underpinnings (e.g. ontological and epistemological assumptions; guiding theoretical framework(s)) clear, consistent and conceptually coherent?** |
| Somewhat   - The authors have not explicitly stated any underpinnings; however, they have analysed their data using grounded theory techniques and have referenced Strauss and Corbin. The process they followed for analysis is in line with grounded theory. |

Padgett et al.^32^

| **1. Was there a clear statement of the aims of the research?** |
| --- |
| No |
| **2. Is a qualitative methodology appropriate?** |
| Can’t tell |
| **3. Was the research design appropriate to address the aims of the research?** |
| Can’t tell   - Due to the confusion with the aims, it is not possible to say with confidence whether the research design is appropriate. |
| **4. Was the recruitment strategy appropriate to the aims of the research?** |
| No   - There is no information on how potential participants were identified and approached. - The authors interviewed 57 participants from the total sample of 184 participants. The authors stopped at 57 interviews because they did not have the resources to undertake all 184 interviews. They do not consider that the data generated through 184 interviews would likely have been very (too) difficult to qualitatively analyse. - There is no explanation of how or why the 57 participants were selected from the total sample of 184 participants. - 57 participants were interviewed and 8 of these were removed from the analysis because of missing data, but it is not clear what constituted missing data in this context or why it was missing. |
| **5. Was the data collected in a way that addressed the research issue?** |
| No   - There is very brief information about the topic guide. The line of questioning (open-ended questions about women’s experiences and responses to abnormal results and recommendations for follow up) seems related to the aims mentioned in the introduction, but less so for the (different) aims mentioned in the methods. |
| **6. Has the relationship between researcher and participants been adequately considered?** |
| Yes   - All interviews were conducted in settings chosen by the interviewees. There was interview training provided for the interviewers, who were instructed to be empathic, non-judgemental listeners and to use sensitive, non-directive probes. - Interviewers of the same minority ethnic background as the interviewees were chosen. |
| **7. Have ethical issues been taken into consideration?** |
| Somewhat   - Ethical approval has been granted by a committee, but there is a lack of evidence in the report that ethical standards were maintained throughout the course of the research. |
| **8. Was the data analysis sufficiently rigorous?** |
| No   - The data analysis and results section is poor. - There is some description on the process of applying codes and developing a code list. There is no explanation for how these codes were then organised into themes. - Parts of the narrative are not supported by data. In places, the data is left to speak for itself. - Parts of the narrative are descriptively thin and, as such, appear unfinished/underdeveloped. Themes 3 and 4 are particularly brief. For example, theme 3 is four lines long (42 words), suggesting that there may not have been enough data for it to be a standalone theme. - Parts of the narrative are not narrative, but are sentences that would have been better placed in the Introduction or the Discussion e.g. discussion of the utility of ultrasound. |
| **9. Is there a clear statement of findings?** |
| No   - There is no clear statement of findings. The authors do not explicitly relate their findings back to their research aims. There is little to no discussion of the evidence for and against the researchers’ conclusions. |
| **10. How valuable is the research?** |
| This research is of poor quality. The authors have not adequately discussed the research limitations. The authors make a number of practice and research recommendations, but these are not always supported by their results. The authors run the risk of overstating their findings by making recommendations with the quality of analysis and results to support them. |
| **11. Are the study’s theoretical underpinnings (e.g. ontological and epistemological assumptions; guiding theoretical framework(s)) clear, consistent and conceptually coherent?** |
| No   - The study’s underpinnings have not been clearly reported. The authors state they are using techniques linked to grounded theory (e.g. line by line coding), but the quality of the analysis is poor, suggesting grounded theory approaches have not been applied effectively. For example, the narrative does not feel grounded in the data. |

Solbjor et al.^30^

| **1. Was there a clear statement of the aims of the research?** |
| --- |
| Somewhat |
| **2. Is a qualitative methodology appropriate?** |
| Yes |
| **3. Was the research design appropriate to address the aims of the research?** |
| Yes |
| **4. Was the recruitment strategy appropriate to the aims of the research?** |
| Yes   - The authors wanted to capture ‘real time’ experiences and as a result were quick to recruit women at the point of being recalled. There is a detailed description of how this was achieved. |
| **5. Was the data collected in a way that addressed the research issue?** |
| Somewhat   - Data collection is clearly described. The authors conducted semi-structured interviews at two times points (just being screening assessment and just after assessment/final results) to explore ‘real time’ experiences of being recalled. - There is a lack of information about the topic guides. This would have been helpful, particularly as the aims of the research are somewhat vague. It would have been interesting to see how the two interview topic guides differed. |
| **6. Has the relationship between researcher and participants been adequately considered?** |
| Yes   - The authors have considered the relationship between researcher and participant. Further, the authors describe how the participants responded to the researcher too. The dynamic has been described. |
| **7. Have ethical issues been taken into consideration?** |
| Somewhat   - The research gained ethical approval by a medical research ethics committee. However, there is little mention of maintaining ethical standards throughout the research, e.g. there is no mention of obtaining informed consent for either interview. Two participants withdrew between interviews, but there is no explanation of why this may have been. - The authors recognise that conducting interviews so close to the recall examination may have been distressing for participants, and a couple of participants withdrew from the study, but the authors do not appear to have taken any steps to support participants. They have stated that such effects are difficult to avoid in a real-time design. |
| **8. Was the data analysis sufficiently rigorous?** |
| No   - Themes were not always developed from codes – the authors report five ways through which they developed themes; coding is one of these five approaches. - Of concern, some themes were developed “through the data […] when anticipated issues were notably missing”, implying some themes were developed from an absence of expected data. - Some themes were anticipated on the basis of theory and earlier research, but which theories or existing pieces of research that contributed to this, and how, is not explained. - The final themes were then compared back to the interview transcripts, wherein emotions, body language and meta-language were also considered. What this involved or how the authors had this data (if it was data) is not clear. |
| **9. Is there a clear statement of findings?** |
| No   - There is no summary or statement of findings. - There is little to no discussion of methodological limitations. The authors claim they cannot generalise their findings because of a small sample – this is problematic as (a) qualitative research does not try to generalise findings and (b) small sample sizes are perfectly find for qualitative research. |
| **10. How valuable is the research?** |
| There is an interesting discussion of existing and relevant literature, but this could have been better linked to the findings. The authors make one clear recommendation – that more information is needed so that women can make more informed decisions when weighing up the pros and cons of screening – and particularly “research is warranted about the kind of information that people are able to absorb during screening, and at which points of the screening trajectory information is important”, which offers good understand of the potential issues of giving more information to women. However, the data analysis is arguably poor and any research or practice recommendations should be considered with this in mind. |
| **11. Are the study’s theoretical underpinnings (e.g. ontological and epistemological assumptions; guiding theoretical framework(s)) clear, consistent and conceptually coherent?** |
| No   - The authors combine their positions - “our experiences and metatheoretical identities vary across a range of QL approaches – phenomenology, grounded theory, discourse analysis – and our work on this project does not represent a clear commitment to any one of these but rather to a mixture of the three.”. However, these approaches are considered to be quite diverse and incompatible, and the analysis they describe is not clearly aligned with any of the three. The authors report their backgrounds briefly and state that the first author read and coded all the interviews – it would have been useful to know the position of this author. |

Thomson et al.^33^

| **1. Was there a clear statement of the aims of the research?** |
| --- |
| Yes |
| **2. Is a qualitative methodology appropriate?** |
| Yes |
| **3. Was the research design appropriate to address the aims of the research?** |
| Somewhat   - The second aim (to explore perceptions of the value, risks and benefits associated with mammography within the false positive context) is not addressed by the research design. |
| **4. Was the recruitment strategy appropriate to the aims of the research?** |
| Yes   - The recruitment strategy has been described well. The eligibility criteria and the process of identifying potential participants are described clearly. - The authors have reported the response rate and statistically examined differences between those women who did and did not respond. - The authors recruited women who had received a false positive screening test results within 3 months, which is appropriate for their research aims. |
| **5. Was the data collected in a way that addressed the research issue?** |
| Somewhat   - The collected data has not addressed the second aim (second research issue) particularly well. Data to answer the first aim was collected through semi-structured interviewers and the topic guide has been described well, but the interview did not cover the second aim. Instead, to address the second aim, women were given written materials by the National Cancer Institute that summarised the risks and benefits associated with mammography. The women were then asked to explain the materials in their own words to the researchers, to demonstrate their understanding, and were then asked for their opinion on the materials. However, it may have been more beneficial to ask women’s perceptions of the value, risks and benefits of mammography, *before* showing them these materials and subsequently repeating the exercise after women had read the materials. |
| **6. Has the relationship between researcher and participants been adequately considered?** |
| No   - There is no evidence that the researcher examiner their own role, potential bias or influence during the formulation of the research question, data collection (including recruitment), or analysis. |
| **7. Have ethical issues been taken into consideration?** |
| Somewhat   - The authors obtained ethical approval from an Institutional Review Board. There is little detail on maintaining ethical standards throughout the course of the research. |
| **8. Was the data analysis sufficiently rigorous?** |
| No   - The authors have used directed content analysis to analyse their data but have not described the process of analysis. They have provided a reference, but this does not adequately describe the process either. - The initial coding categories were derived from the literature, but how these categories were chosen is not clear. The references provided are for systematic reviews of false positive test results in mammography, but how this research led to or provided qualitative coding categories has not been explained. - There are some issues with the narrative and themes. The narrative is generally detailed and well-balanced (negative cases have been presented), but some parts of the narrative do not appear to be related to the overarching category or theme, and the themes do not always relate back to the research question. Data excerpts do not always support the narrative; there is some indication that the authors have overinterpreted their data. - The authors use %’s to describe the proportion of women in the sample that held a particular opinion, but it is not clear whether it is a % of the sample after every woman was asked to give her opinion, or whether it is a % of women who spontaneously shared a particular opinion. |
| **9. Is there a clear statement of findings?** |
| Somewhat   - There is a reasonably clear statement of findings. - Some of the findings are at risk of being overstatements and, as a result, the implications drawn from these findings are at risk too. - The authors have made some attempt to discuss the reasons for and against their argument and conclusions. |
| **10. How valuable is the research?** |
| The authors have made some attempt to discuss their findings in the context of what is already known. The authors make a reasonable recommendation for research and practice – the need for more information so that women better understand the risks and benefits of mammography. This may well be the case in the USA. However, their second recommendation is that the “screening clinic may be an innovative opportunity to introduce interventions focused [on] additional modifiable risk behaviours…”. This implication must be considered with caution as the theme upon which it is based is of questionable rigour. |
| **11. Are the study’s theoretical underpinnings (e.g. ontological and epistemological assumptions; guiding theoretical framework(s)) clear, consistent and conceptually coherent?** |
| No   - The authors have not explicitly stated their underpinnings. - The authors state that they used directed content analysis, with no real explanation of what this involved, but have applied grounded theory techniques. |

**Supplementary materials 5. Thematic map of findings, showing the relationship between first order codes, second order codes, descriptive themes and analytical themes**

| **Analytical themes** | | |
| --- | --- | --- |
| Expectations for their health and screening experience | Living with uncertainty | Restoration of the healthy self |

| **Descriptive themes** | | | | | | | |
| --- | --- | --- | --- | --- | --- | --- | --- |
| Perceptions of health | State of uncertainty | Making sense of the situation | Being ‘looked after’ by the system | Attitudes towards screening and re-attendance | Breast cancer specific worries and anxieties | Complying with the screening programme | Reappraisal of life |
| **Codes** | | | | | | | |
| -Perceived self as healthy prior to recall (1^st^*,2^nd^**)  -Screening as means to validate perceived good health (1^st^,2^nd^)  -Being recalled introduces doubts about healthy self (1^st^,2^nd^)  -Reason for recall = something wrong with self (internal)/equipment (external) (2^nd^)  -Forced shift in perception of self to include BC (2^nd^)  -Assumed ‘the worst’ (1^st^,2^nd^)  -Recall letter interpreted as cancer (1^st^,2^nd^)  -Mentally preparing for cancer diagnosis (1^st^)  -Image of lesion interpreted as cancer (1^st^)  -Experience a diagnosis of cancer (2^nd^)  -Identifying with patients with cancer (2^nd^)  -Imagining self/life with cancer (2^nd^)  -Health reaffirmed with final result (1^st^,2^nd^) | -State of uncertainty about health (2^nd^)  -‘in limbo’ until final result (1^st^, 2^nd^)  -No choice but to ‘live’ the waiting time (2^nd^)  -Uncertainty is stressful (2^nd^)  -Uncertainty is difficult to live with (2^nd^)  -Final result ends period of uncertainty/offers certainty (1stn, 2^nd^)  -Trust in HCPs judgement and final result (2^nd^_  -Trust in accuracy of final result (2^nd^)  -Poor interpersonal communication with consultant contributes to uncertainty (2^nd^)  -Ambiguous explanations contribute to lasting uncertainty (1^st^, 2^nd^)  -Uncertainty in ‘just not knowing’ (1^st^)  -Unclear or inadequate explanation contributes to lasting uncertainty (1^st^, 2^nd^,)  -Unanswered questions about reasons for recall (1^st^) | -Attempt to be rational/realistic about risk (1^st^, 2^nd^)  -Attempt to dismiss cancer as a possibility (1^st^)  -Adopt a ‘wait and see’ approach (1^st^)  -Increased breast self-examination (1^st^, 2^nd^)  - Difficulty understanding risk estimates (1^st^, 2^nd^)  -Drawing on the recall experiences of others (1^st^, 2^nd^)  -Drawing on previous screening experiences (1^st^, 2^nd^)  -Seeking examples of other women’s experiences (1^st^, 2^nd^)  -Reassured by wording of recall letter (2^nd^)  -Reassured by positive messages in recall letter (1^st^, 2^nd^)  -Unmet information needs (1^st^)  -Information given in recall letter varies and inconsistent (2^nd^)  - FHBC = reassuring or worrying (1^st^, 2^nd^)  -Interpreting communication between HCPs to gauge risk/result (1^st^, 2^nd^)  -Being seen quickly = something wrong (1^st^, 2^nd^) | -Welcomed the invitation (1^st^, 2^nd^)  -Relieved to be invited (1^st^, 2^nd^)  -Screening offers certainty (2^nd^)  -Grateful for the service (1^st^)  -Relieved to be ‘in the system’ (1^st^)  -Being ‘in the system’ offers security and comfort (1^st^, 2^nd^)  -‘looked after’ by the system and HCPs (1^st^, 2^nd^)  -Service as ‘guardian angel’ (2^nd^)  -No longer ‘cared for’ after the service ends (1^st^, 2^nd^)  -Desire to be ‘looked after’ for longer (2^nd^)  -Unmet support needs (1^st^, 2^nd^)  -Supported by family or friends (1^st^, 2^nd^) | -Intend to re-attend (2^nd^)  -Believe in the value and importance of screening (2^nd^)  -Lack of confidence in service (2^nd^)  -Dilemma: fear of cancer vs. fear of screening (2^nd^)  -The harms of screening: a catch-22 (2^nd^)  -The worry is worth it (2^nd^)  -Motivated to re-attend to get treatments for early cancer (2^nd^)  -Worry creates need for more screening and more reassurance (2^nd^)  -Doubts about active re-attendance after programme ends (1^st^, 2^nd^)  -Desire to be screened (for longer) (1^st^)  -No desire for more screening but common sense to go (1^st^)  -Better safe than sorry (1^st^)  -Desire for shorter intervals between screenings (1^st^)  -Doubts about re-attendance: is it worth it? (1^st^, 2^nd^)  -Decision whether to re-attend: head vs. heart (1^st^) | -Negative intrusive thoughts (1^st^, 2^nd^)  -Fear of breast cancer (1^st^, 2^nd^)  -Associate breast cancer with death (1^st^, 2^nd^)  -Wording of recall letter creates worry (1^st^)  -Worried about impact (of breast cancer/death) on family (1^st^, 2^nd^)  -Worried about consequences of breast cancer (1^st^, 2^nd^)  -Anxiety about recall appointment (2^nd^)  -Anxiety about results (having breast cancer) (1^st^, 2^nd^)  -Anxiety in the waiting room (2^nd^)  -Anxiety regarding future screenings and results (2^nd^)  -Not worried by recall; confidence in health (2^nd^)  -Image of lesion triggers anxiety (about having breast cancer) (2^nd^)  -Overcome with emotions after final result (in appt.) (2^nd^)  -Lingering breast cancer worries (1^st^)-Time should be a healer (but is not) (1^st^)  -Subsequent screening invitations trigger worry (1^st^) | -Screening is part of health care routine (2^nd^)  -Attending screening = behaving responsibly (1^st^, 2^nd^)  -Recall appointment and tests = behaving responsibly (passive) (2^nd^)  -Passive service user (1^st^, 2^nd^)  -Perceived lack of patient choice (disempowered) (1^st^, 2^nd^)  -Desire for more patient choice (1^st^, 2^nd^)  -Attending screening = ‘common sense’ (1^st^)  -Passivity a result of service set-up (1^st^, 2^nd^)  -Felt unable to ask questions to clarify result (2^nd^) | -Relief after final result (1^st^, 2^nd^)  -Grateful, thankful for life (1^st^, 2^nd^)  -More appreciate of life and health (2^nd^)  -Motivated to enjoy life more (2^nd^)  -Considering healthy lifestyle changes (1^st^, 2^nd^)  -Considering personal changes (1^st^, 2^nd^)  -Final result = wake-up call (1^st^) |

*First-order code

**Second-order code

**Supplementary materials 6**

Signs that women interpreted and analysed to gauge their breast cancer risk.

| **Cue** | **Context** |
| --- | --- |
| Their previous screening experiences | Women who had previously had a negative screening test result often took comfort from this, believing the same result was likely this time. Others, who had had a false positive screening test result in the past, described feeling more vulnerable: “[…] you do think, ‘what if there is something this time?’”^7^ |
| The screening experiences of friends and family. | Women reported feeling reassured by the experiences of friends and family who had had a false positive screening test result: “I didn’t really get worried at all. I was kind of like ‘Oh OK’. I’ve had enough friends that have had a similar experience. It’s been nothing. Or my mother… she said that always happens to her.”^33^ |
| The presence or absence of a family history of breast cancer | The presence or absence of a family history of breast cancer was often interpreted as either a worrying or promising sign for their own chance of being diagnosed with cancer: “[…]You know, my mother died from it [breast cancer] when she was 47, so I thought: ‘Well, now I’m probably going to die’. That was my interpretation of the recall letter.”^8^ |
| The wording of the recall letter | Some women reported being reassured by the wording of, and positive messages conveyed by, the recall letter. However, there was variation in the wording of recall letters and information provided; *some* [women] *were simply told they would have another mammogram and others were given an explanation about the reason for their recall.*^29^ Some felt that there was a lack of clarity around what to expect at screening assessment and thus believed they should have been given more information before and during their assessment. If the women were not told explicitly, they did not assume. For example, a missed opportunity to provide information meant the women went to the clinic alone when they would have preferred to have had a supportive companion: “The only thing that I would have liked the letter to have said was, ‘if you want to bring a friend or close relative, please do so.’”^29^ |
| The risk estimates provided in the recall letter | Some women found the risk estimates provided in the recall letter reassuring, believing the odds were in their favour.^29^ Others found them worrying and difficult to interpret.^30^ Women recognised that risk assessments had been provided to reassure them, but generic and impersonal estimates were not especially reassuring: *the objectivity of numerical estimates vanished when relating them to oneself.*^30^ |
| The location of their screening assessment | Some women had their initial screening mammogram in a mobile unit, and were concerned that their screening assessment was taking place in a hospital, for this seemed more serious. |
| The turnaround time between their recall letter and screening assessment | Being seen quickly for screening assessment was perceived as a sign of urgency and of something seriously wrong. Others appreciated the fast turnaround because it minimised the time spent worrying. |
| The communication with and between HCPs | Women read between the lines of what the HCPs were saying and doing, e.g. an arrangement to receive results via the telephone, rather than in a hospital visit, was reassuring: “[…] after all, she wouldn’t arrange a phone call if she knew it was dangerous. Otherwise, she would have wanted to meet me face-to-face.”^5^ |
| The image of their lesion | Some were convinced that they were seeing unambiguous, visual evidence of breast cancer, but others were surprised to have been recalled for something: “[…] you could hardly see […]”.^7^ |
| Breast self-examination | Women reported increased breast self-examination to feel for breast abnormalities. |

*Table 1.* Eligibility criteria

| Inclusion criteria |
| --- |
| 1. Qualitative methodology (i.e. data collection and analysis). |
| 1. Adult women (aged 18+ years) who have received a false positive breast screening result or an abnormal breast screening result (and are awaiting screening assessment or the associated results). |
| 1. Mixed samples of adults screened for, or diagnosed with, other cancer types only if it is possible to separately identify those findings related to having a false positive breast screening result. |
| 1. Any country. 2. Published in English. |
|  |
| Exclusion criteria |
| 1. Study findings could not be separated in criterion (c). |
| 1. The sample was all diagnosed with breast cancer (invasive and ductal carcinoma in situ). |
| 1. Individual case studies. |

*Table 2*. Main characteristics of included papers.

| **Author (year)** | **Country** | **Participants** | **Time since false positive test result** | **Data collection** | **Data analysis** |
| --- | --- | --- | --- | --- | --- |
| Bolejko et al. (2014) | Sweden | n=13  Age range 40 to 68y  Mean age 51y | Range: 3-11m | Semi-structured interviews | Inductive content analysis |
| Bond et al. (2015a) | UK | n=21  Age range 42 to 69y  Mean age 59y | ≤1y n=4  2–4y n=7  5–7y n=8  8–10y n=1  11–13y n=1  Mean: 4.4y | Semi-structured interviews | Interpretative phenomenological analysis |
| Bond et al. (2015b) | UK | n=21  Age range 42 to 69y  Mean age 59y | ≤1y n=4  2–4y n=7  5–7y n=8  8–10y n=1  11–13y n=1  Mean: 4.4y | Semi-structured interviews | Inductive content analysis |
| Fielding & Lam (2007) | Hong Kong | n=9  Age range 40 to 62y | Not reported | Semi-structured interviews | Not explicitly aligned with one method; appears to be thematic |
| Lindberg et al. (2013) | Denmark | n=8  Age range 57 to 72y  Mean age 65y | Range: 4-5y | Semi-structured interviews | Grounded theory |
| Padgett et al. (2001) | USA | n=45  Mean age 52y | Not explicitly reported; at least 6-8m | Interviews with open ended questions | Not explicitly aligned with one method; describes grounded theory techniques |
| Solbjor et al. (2011) | Norway | n=8  Age range 50 to 59y | Not yet received false positive test result; interviewed the day before screening assessment | Semi-structured interviews at two time points | Not explicitly aligned with one method; appears to be thematic |
| Thomson & Siminoff (2015) | USA | n=40  Age range 40 to 68y | <3m | Interviews with open ended questions and questions about reactions to health guidelines | Directed content analysis |

*Table 3*. Signs that women interpreted and analysed to gauge their breast cancer risk.

| **Sign** |
| --- |
| Their previous screening experiences.  The screening experiences of friends and family.  The presence or absence of a family history of breast cancer.  The wording of the recall letter.  The risk estimates provided in the recall letter.  The location of their screening assessment.  The turnaround time between their recall letter and screening assessment.  The communication with and between HCPs.  The image of their lesion.  Breast self-examination. |


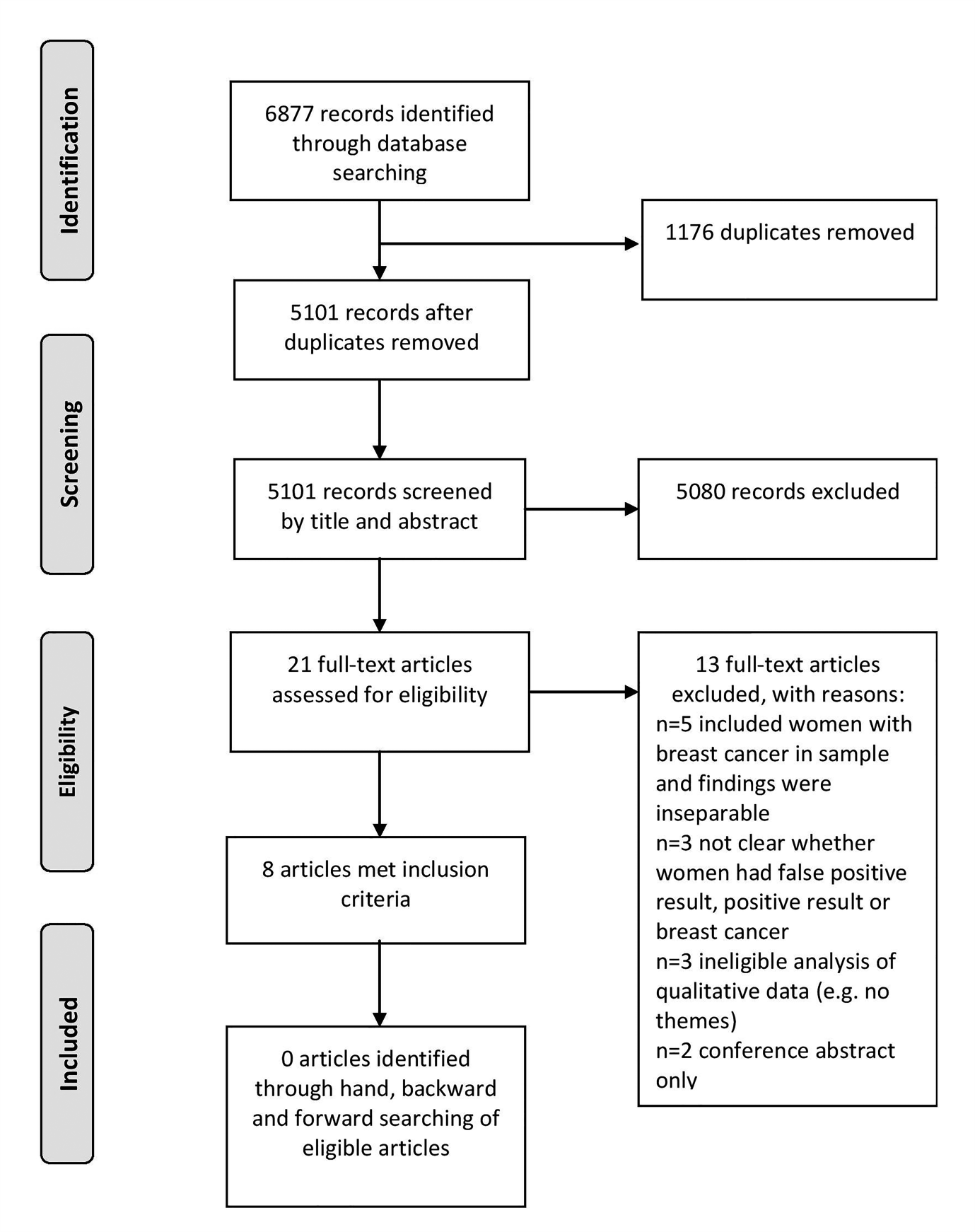


*Figure 1.* Flow diagram of study inclusion process.
